# A viral ubiquitination switch attenuates innate immunity and triggers nuclear import of virion DNA and infection

**DOI:** 10.1126/sciadv.abl7150

**Published:** 2021-12-17

**Authors:** Michael Bauer, Alfonso Gomez-Gonzalez, Maarit Suomalainen, Nicolas Schilling, Silvio Hemmi, Urs F. Greber

**Affiliations:** 1 Department of Molecular Life Sciences, University of Zurich, Winterthurerstrasse 190, Zurich CH8057, Switzerland.; 2 Life Science Zurich Graduate School, ETH and University of Zurich, Zurich 8057, Switzerland.; 3 Center for Microscopy and Image Analysis, University of Zurich, Winterthurerstrasse 190, Zurich CH-8057, Switzerland.

## Abstract

Antiviral defense and virus exclusion from the cell nucleus restrict foreign nucleic acid influx and infection. How the genomes of DNA viruses evade cytosolic pattern recognition and cross the nuclear envelope is incompletely understood. Here, we show that the virion protein V of adenovirus functions as a linchpin between the genome and the capsid, thereby securing particle integrity. Absence of protein V destabilizes cytoplasmic particles and promotes premature genome release, raising cytokine levels through the DNA sensor cGAS. Non-ubiquitinable V yields stable virions, genome misdelivery to the cytoplasm, and increased cytokine levels. In contrast, normal protein V is ubiquitinated at the nuclear pore complex, dissociates from the virion depending on the E3 ubiquitin ligase Mib1 and the proteasome, and allows genome delivery into the nucleus for infection. Our data uncover previously unknown cellular and viral mechanisms of viral DNA nuclear import in pathogenesis, vaccination, gene therapy, and synthetic biology.

## Teaser

The E3 ubiquitin ligase Mib1 and proteasome promote infection by releasing the linchpin protein V from incoming adenovirus.

## INTRODUCTION

Viruses coevolve with their hosts in entry, uncoating, replication, assembly, and innate and adaptive immunity (*1*, *2*). Their DNA or RNA genomes give rise to two opposing reactions, viral gene expression driving infection and innate immune response restricting infection through activation of DNA and RNA sensors. Viral gene expression requires uncoating of the incoming genome, while innate immunity is enhanced by premature genome exposure leading to abortive infection.

Many DNA viruses, including adenoviruses (AdVs), bring their genome into the cell nucleus driving viral transcription and progeny formation (*3*). Early viral gene products counteract the innate immune response against the incoming virus, for example, the E1A and E1B-55K proteins of AdV blunt the interferon (IFN) response (*4*–*7*). This is important because pathogen-associated molecular patterns (PAMPs) activate the innate immune system as early as pathogen entry and boost cell defense and adaptive immunity (*8*). PAMPs are decoded by pattern recognition receptors, for example, Toll-like receptors, DNA and RNA sensors such as cyclic guanosine monophosphate (GMP)–adenosine monophosphate (AMP) synthase (cGAS), nucleotide-binding oligomerization domain–like receptors driving inflammasome formation, retinoic acid–inducible gene I–like receptors, or cytosolic lectins, and can trigger inflammatory responses (*9*, *10*). The extent and duration of the innate immune response are determined by autoregulation processes and the nature of the PAMPs.

We show here that AdV sequesters double-stranded viral DNA (vDNA) PAMPs from cytosolic sensors through the virion protein V. The vDNA core contains two major basic proteins, a histone-like organizer protein VII and a linker protein V, both of which bind the DNA in a sequence-independent manner. Protein VII provides basic nuclear localization sequences for nuclear import of the vDNA and reduces the accessibility of the vDNA to the DNA damage recognition machinery (*11*–*14*). It remains associated with the vDNA until transcription in the nucleus (*15*–*17*), unlike protein V, which bridges the DNA core and the capsid, and dissociates from the virion before nuclear import of the genome (*18*).

Here, we demonstrate that ubiquitination of protein V dislocates protein V from the vDNA upon virion disruption at the nuclear pore complex (NPC), a process known to require the coordinated action of microtubules and the microtubule motor kinesin-1 (*19*). Protein V is a highly conserved, lysine-rich capsid protein present in all mammalian AdVs across 51 species and more than 100 human types (http://hadvwg.gmu.edu/). Lysine residues on a plethora of viral and cellular proteins are ubiquitinated by more than 600 different E3 ubiquitin ligases affecting protein stability and signaling in diverse processes, including endocytosis, transcription, the cell cycle, and virus infection (*20*, *21*). Viruses take advantage of the ubiquitin-proteasome system (UPS) to degrade host factors that restrict their replication. In addition, cellular and viral ubiquitin and ubiquitin-like proteins as well as E3 ligases and adaptors for ligase recruitment play important roles, as shown with the human papillomavirus E6 and E7 proteins, the AdV E1B-55K/E4Orf6 complex, or the herpes simplex virus type 1 ICP0 (infected cell protein 0) protein targeting the tumor suppressor protein p53 [reviewed in (*22*, *23*)]. Ubiquitination has also been observed in viral proteins during entry, for example, with influenza virus and AdV (*24*, *25*). UPS mechanisms in virus stability and genome uncoating, however, have remained largely elusive.

Here, we elucidate how AdV secures its genome in the protein capsid while in transit through the cytoplasm and then lays it open for nuclear import and transcription. Human AdVs are widespread non-enveloped agents causing some 5% of all respiratory infections (*26*). AdV infections occur throughout the year, sporadically and sometimes epidemically, mostly self-limiting in the respiratory, ocular, and gastrointestinal tracts, where virus persists for many years and gives rise to long-term bronchiectasis and bronchiolitis obliterans and increases the risk of protracted bacterial bronchitis due to chronic neutrophilic inflammation (*26*–*28*). AdVs reactivate during episodes of compromised immunity, e.g., after organ transplantation or anti-inflammatory treatment of autoimmune disease, and cause viremia and life-threatening conditions with morbidity and mortality, highlighting the importance of immune control, including IFN (*28*, *29*). In addition, human and chimpanzee AdVs have a long history as vectors in gene therapy and vaccination, including the prevention of COVID-19 caused by SARS-CoV-2 (*30*–*32*).

Using viral and cellular mutagenesis, mass spectrometry (MS), quantitative fluorescence microscopy in live and static modes, DNA click chemistry, electron microscopy of high-pressure frozen (HPF-EM), freeze-substituted samples, and cytokine profiling, we uncover that protein V secures the viral genome in transit through the cytoplasm and detaches from the genome upon ubiquitination depending on the RING-type E3 ligase Mind bomb 1 (Mib1). Mib1 transfers polyubiquitin chains with several different linkages, traffics on centriolar satellites, and is involved in Notch pathway signaling and ciliogenesis (*33*, *34*). Cells lacking Mib1 or expressing ubiquitination-defective Mib1 deadlock AdV capsids at the NPC (*35*, *36*). Our data here uncover that a virion linchpin protein V suppresses PAMPs and is inactivated by Mib1 and the UPS at the NPC, thereby triggering vDNA uncoating, nuclear import, and infection.

## RESULTS

### Phenotypically normal and entry-competent virions lacking protein V

**Fig. 1.**
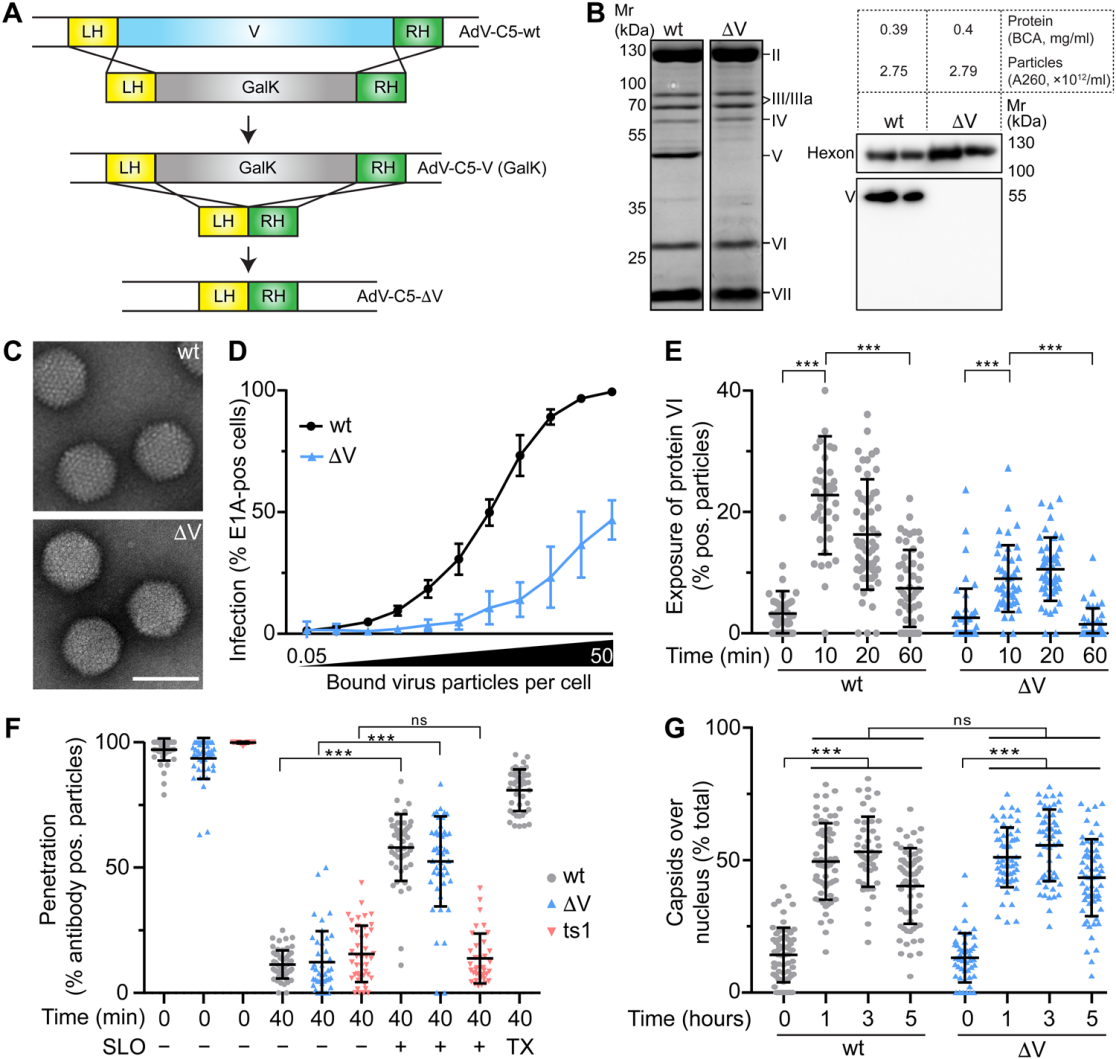
Viral particles lacking protein V are less infectious than the wild-type particles. (**A**) GalK-based recombineering strategy for AdV-C5-ΔV using left homology (LH) and right homology (RH) arms of 45 base pairs up- or downstream of the protein V coding region. wt, wild type. (**B**) Protein composition of AdV-C5 and AdV-C5-ΔV particles. SDS-PAGE/QuickBlue staining (left) and Western blot immunostained for hexon and protein V (right). Apparent molecular weights and viral proteins are indicated. For both virus preparations, protein concentration [measured by bicinchoninic acid (BCA) assay] and number of virus particles (based on the absorbance at 260 nm) are indicated. (**C**) Electron micrographs of negatively stained AdV-C5 or AdV-C5-ΔV particles. Scale bar, 100 nm. (**D**) AdV-C5 and AdV-C5-ΔV infections of HeLa cells using 0.05 to 50 viral particles per cell upon cold binding, followed by infection at 37°C for 20 hours, and anti-E1A antibody staining. Data represent means ± SD of the E1A-positive 4′,6-diamidino-2-phenylindole (DAPI)–stained nuclei. (**E**) Analysis of protein VI exposure. Virus was cold-bound and internalized to A549 cells, and cells were stained with anti-hexon 9C12, anti-pVI, DAPI, and Alexa Fluor 647–conjugated succinimidyl ester. Cell-associated virus particles were segmented on the basis of hexon signal. Statistical significance determined by nonparametric analysis of variance (ANOVA) (Kruskal-Wallis test) with Dunn’s correction for multiple comparisons. ***P < 0.001; ns, not significant. (**F**) Penetration of incoming particles into cytoplasm. A549 cells were cold-bound with ATTO565-labeled AdV-C5, AdV-C5-ΔV, or Alexa Fluor 488–labeled AdV-C2 ts1, washed, and incubated at 37°C for 0 or 40 min, permeabilized with SLO, incubated with anti-hexon or anti–Alexa 488 antibodies, fixed, and stained with secondary antibodies, DAPI, and Alexa Fluor 647–conjugated succinimidyl ester. Triton X-100 addressed the accessibility of the antibodies. Statistical significance as in (E). (**G**) Nuclear targeting of incoming AdV-C5 and AdV-C5-ΔV particles. AdV-C5 or AdV-C5-ΔV was cold-bound to A549 cells, fixed or incubated at 37°C, and stained with anti-hexon, DAPI, and Alexa Fluor 647–conjugated succinimidyl ester. Virus particles were segmented and masked with the nucleus. Statistical significance as in (E).

Despite decades of molecular, structural, and translational research in AdV, the function of one prominent virion protein, protein V, present in all mastadenoviruses has remained unknown, although some features of protein V have been reported (*31*). It binds to DNA, and its mass makes up nearly 30% of the proteins in the core with 50% vDNA and 50% protein mass. Protein V has been suggested to be redundantly involved in virion assembly, being sumoylated during replication and bridging the core and the capsid wall by interacting with protein VI, thereby stabilizing the capsid (*37*, *38*). To determine whether protein V affects virus entry into host cells, we generated an AdV-C5 mutant with a deletion of the protein V coding region (AdV-C5-∆V) (Fig. 1A). Particles were isolated by cesium chloride gradient centrifugation and analyzed by SDS–polyacrylamide gel electrophoresis (SDS-PAGE). QuickBlue staining and Western blotting showed a lack of protein V but no differences in the major virion proteins, including hexon, penton base, IIIa, fiber (IV), VI, and VII (Fig. 1B). Proteins VIII, IX, X, and IVa2 and protease were not visualized in these gels. Sanger sequencing confirmed that the protein V coding region was removed from the viral genome (fig. S1A). Full analyses of the vDNA by next-generation sequencing revealed no other mutations in the viral genome (fig. S1B and data S1). Last, the integrity of the virions was verified by transmission electron microscopy of negatively stained specimens (Fig. 1C). To test whether protein V was involved in single-round infections, we measured the immediate early viral protein E1A at 20 hours post infection (pi). Inocula of AdV-C5-∆V and the parental AdV-C5 wild type were adjusted to the number of particles bound per cell, as previously described (*39*). To reach a similar level of E1A expression, a considerably higher number of AdV-C5-∆V particles were required per cell, as compared to the wild type, indicating that particles lacking protein V were less infectious (Fig. 1D). Analyses of single fluorescent particles using confocal light microscopy revealed similar kinetics in the exposure of the endosome lytic protein VI and no difference in viral escape from the endosomes or trafficking to the nucleus between AdV-C5-∆V and wild-type AdV-C5 (Fig. 1, E to G).

**Fig. 2.**
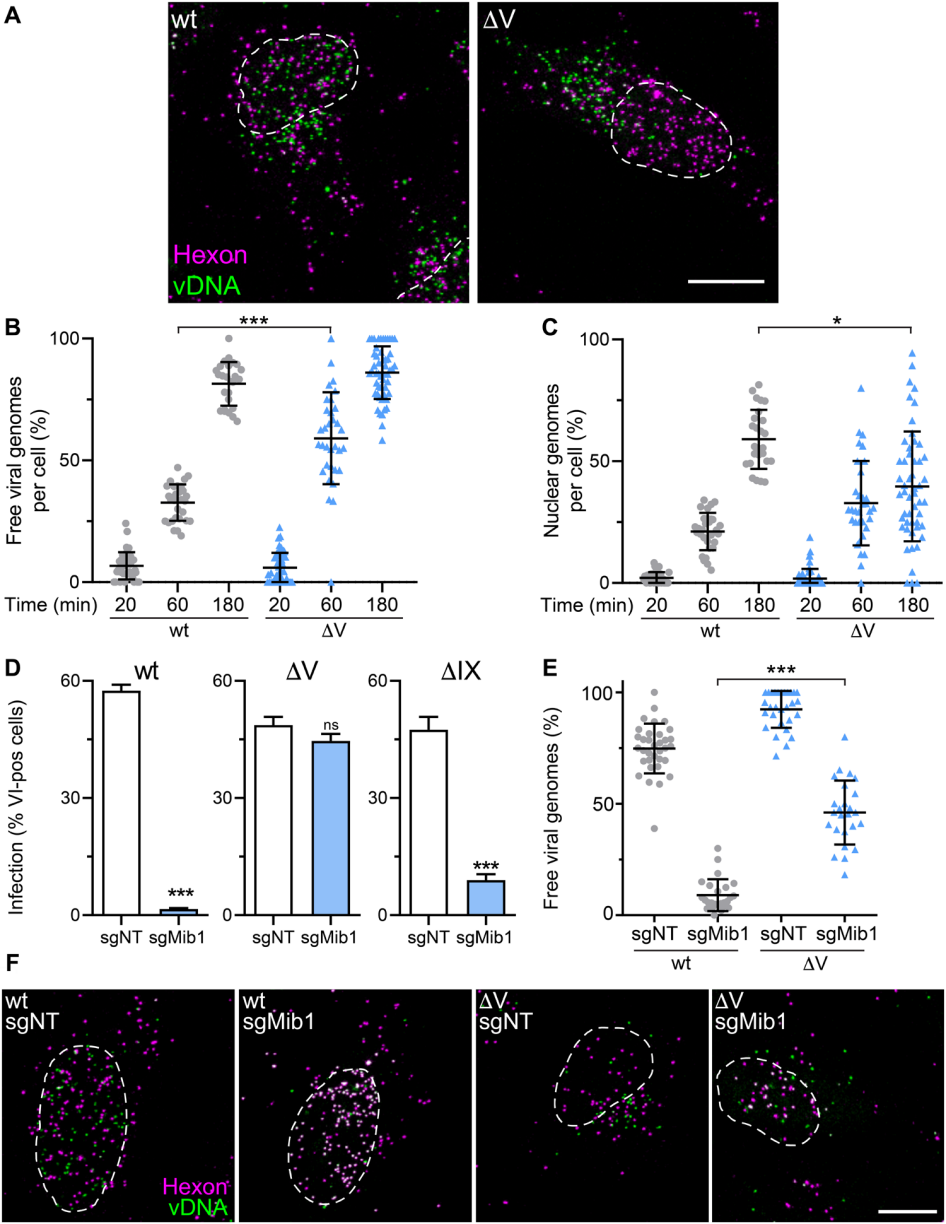
AdV-C5-ΔV particles deliver higher amounts of vDNA to the cytoplasm and are less dependent on Mib1 than wild-type AdV. (**A**) Uncoating of AdV-C5 and AdV-C5-ΔV particles. HeLa cells were incubated with genome-labeled AdV-C5 or AdV-C5-ΔV for 3 hours and fixed; capsids were immunostained with anti-hexon antibodies, and viral genomes were visualized by click chemistry with an N3 Alexa Fluor 488. Other details as in Fig. 1G. Scale bar, 10 μm. (**B** and **C**) Quantification of genome uncoating. HeLa cells were incubated with genome-labeled AdV-C5 or AdV-C5-ΔV for 20, 60, or 180 min and processed as in (A). Viral genomes were segmented and classified as capsid-free on the basis of their hexon intensity. Genomes over the nuclear mask were counted as nuclear. Each dot represents one cell. Data are shown as means ± SD. Statistical significance was assessed as described in Fig. 1E. *P < 0.05 and ***P < 0.001. (**D**) Infection efficiencies of AdV-C5, AdV-C5-ΔV, and AdV-C5-ΔIX particles. Control HeLa-sgNT and Mib1-KO (sgMib1) cells were infected with the indicated viruses at a MOI of 0.4. After 24 hours, cells were fixed and stained with anti–protein VI antibodies. Nuclei were segmented on the basis of DAPI signal, and infection measurement was based on protein VI signal. Error bars represent means ± SD. *P* values were assessed using unpaired *t* tests. ***P < 0.001. (**E** and **F**) Uncoating of AdV-C5 and AdV-C5-ΔV particles in control and Mib1-KO cells. HeLa-sgNT and sgMib1 cells were incubated with genome-labeled AdV-C5 or AdV-C5-ΔV for 3 hours, fixed, and stained with anti-hexon and vDNA using click chemistry. Viral genomes were quantified as in (B). Statistical significance was assessed as described in Fig. 1E. ***P < 0.001. Scale bar, 10 μm.

Closer analyses of incoming viral genomes tagged with 5-ethynyl-2′-deoxycytidine (EdC) nucleotides (*17*, *35*) revealed significant differences between AdV-C5-∆V and the wild type. Nearly all genomes were released from both wild-type and AdV-C5-∆V capsids at 3 hours pi (Fig. 2, A and B). However, most wild-type genomes were delivered to the nucleus, while most of the AdV-C5-∆V genomes were in the cytoplasm (Fig. 2, A to C). This defect in nuclear import of AdV-C5-∆V DNA likely explains the low infectivity of these particles.

Nuclear import and uncoating of AdV depends on the E3 ubiquitin ligase Mib1, and absence of Mib1 enriches incoming AdV particles at the NPC (*35*, *36*). We tested whether the absence of protein V affected the infection of Mib1 knockout (KO) cells. Notably, infection of Mib1-KO cells with AdV-C5-∆V but not AdV-C5-∆IX was readily possible, while wild-type infection was blocked (Fig. 2D). A higher number of vDNA molecules were released from AdV-C5-∆V capsids than wild-type AdV in Mib1-KO cells, showing that vDNA release from the AdV-C5-∆V particles was only partially dependent on Mib1 and that this release occurred before docking to the NPC (Fig. 2, E and F).

### AdV-C5-∆V is more thermosensitive and disintegrates in the cytosol, unlike wild-type AdV-C5

**Fig. 3.**
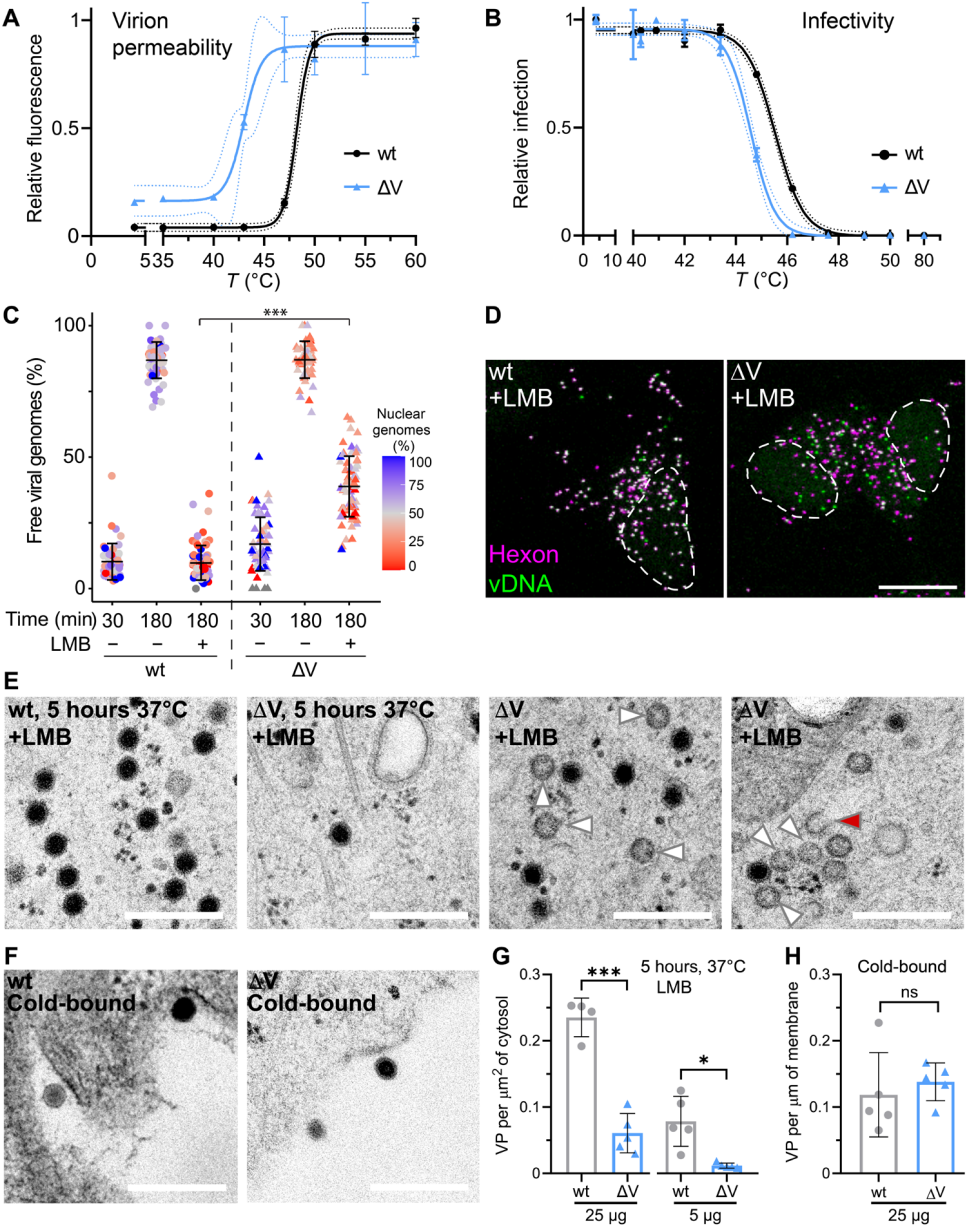
AdV-C5-ΔV particles are less thermostable and release vDNA more frequently than wild-type in the cytoplasm. (**A**) Thermostability of AdV-C5 and AdV-C5-ΔV particles. Virions were kept at 4°C or subjected to indicated temperatures (5 min), cooled down, incubated with DiYO-1 (5 μM, 5 min), and analyzed by fluorimetry. Means ± SD represent data normalized to the maximum fluorescence (60°C). (**B**) Thermostability and infectivity of AdV-C5 and AdV-C5-ΔV particles. A549 cells were incubated with heat-treated virus for 24 hour, fixed, immunostained for protein VI and DAPI, and scored for VI-positive nuclei relative to nontreated virus. Data are means ± SD. (**C** and **D**) Premature uncoating of AdV-C5-ΔV particles in the cytoplasm. HeLa cells were incubated with genome-labeled AdV-C5 or AdV-C5-ΔV with/without LMB (50 nM), fixed, and stained with anti-hexon and vDNA click chemistry. Data are means ± SD. Color-coded percentage of capsid-free genomes over the nucleus. Statistical significance was assessed as described in Fig. 1E. ***P < 0.001. For further details, see Fig. 2A. Scale bar, 10 μm. (**E** and **F**) HPF-EM analyses of AdV-C5– and AdV-C5-ΔV–infected HeLa cells, incubated with AdV-C5 or AdV-C5-ΔV (25 μg each) at 37°C for 5 hours or cold-bound (1 hour, 4°C). White and red arrowheads indicate empty and broken virus particles, respectively. Scale bars, 300 nm. (**G** and **H**) Quantification of HPF-EM experiments. Internalized particle counts were normalized to the cell area and extracellular cold-bound virus to the cell perimeter. Statistical significance from unpaired *t* tests, *P < 0.05 and ***P < 0.001.

To assess the stability of AdV-C5-∆V, we conducted temperature escalation experiments of isolated virions and measured their fluorescence increase in the presence of DiYO-1, a dye that fluoresces upon DNA intercalation and provides a highly sensitive end point assay for the disruption of the capsid shell (*18*). Below 40°C, both wild-type and AdV-C5-∆V particles remained largely intact, as indicated by a low level of DiYO-1 fluorescence, but AdV-C5-∆V readily reached half-maximal fluorescence at 42°C, while wild-type virus was intact up to about 47°C (Fig. 3A). Akin to the disruption profile, the infectivity profile showed higher heat resistance of AdV-C5-wt than ∆V, although the difference was not as pronounced as with the DNA-dye binding assay, suggesting that the ∆V particles become leaky to the dye at 40° to 44°C without losing infectivity, while wild-type particles remain fully intact and infectious (Fig. 3B). This notion was reinforced by single-virus particle analyses in cells, where more than 40% of the incoming AdV-C5-∆V particles lost their genome in the presence of the inhibitor leptomycin B (LMB), a nuclear export inhibitor blocking the transfer of AdV from microtubules to the NPC (Fig. 3, C and D) (*40*). Notably, both wild-type and AdV-C5-∆V infections were equally and strongly inhibited by LMB (fig. S2A). Protein degradation inhibitors, such as DBeQ blocking the p97 adenosine triphosphatase or MLN9708 blocking the proteasome, did not inhibit the premature release of vDNA from AdV-C5-∆V in the presence of LMB (fig. S2, B and C). Likewise, the depletion of microtubules with nocodazole had no effect on genome release and localization of AdV-C5-∆V, suggesting that a tug of war between opposite polarity microtubule motors on cytoplasmic virus particles is not required for premature disruption of AdV-C5-∆V (fig. S2, D and E). Ultrastructural studies by HPF-EM revealed high numbers of empty and broken AdV-C5-ΔV but not wild-type AdV particles in the cytosol (Fig. 3, E to G), while both AdV-C5-ΔV and wild-type AdV particles were equally present at the plasma membrane before internalization (Fig. 3H). Collectively, the results show that AdV-C5-∆V particles are less stable than wild type and suggested that premature release of vDNA from AdV-C5-∆V might be due to intrinsic particle instability in a crowded cytosol.

### Protein V in the virion dampens the cytokine response and innate immunity

**Fig. 4.**
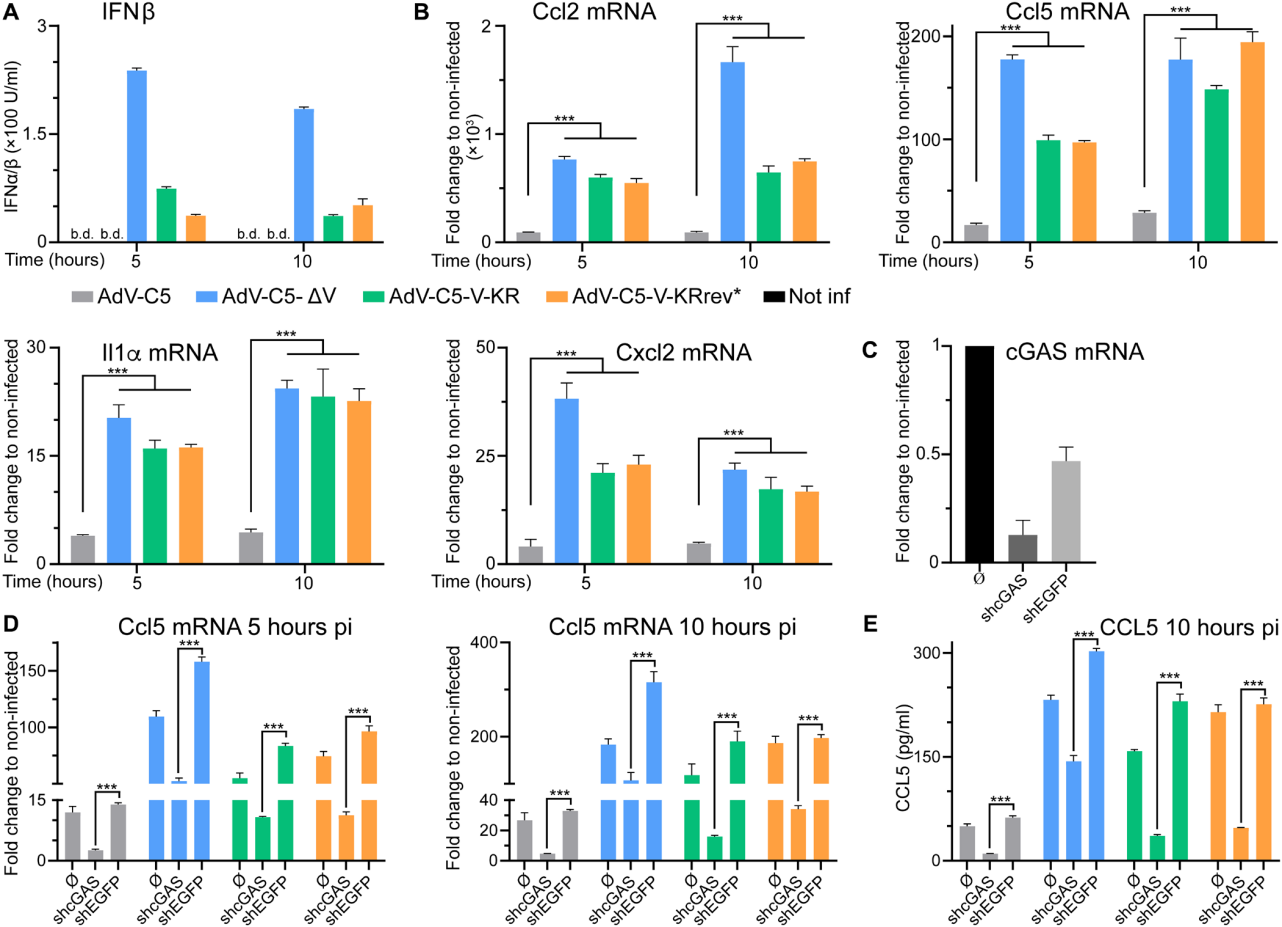
Protein V from incoming AdV suppresses the induction of cytokines during early stages of infection. (**A**) Higher type I IFN levels in macrophage-like MPI-2 cells by mutated protein V than AdV-C5-wt particles. Cells were inoculated with virions (37°C, 30 min), such that about 350 particles were bound per cell, washed, and incubated at 37°C, and supernatant was assayed for type I-IFN. Data represent the luciferase chemiluminescence from reporter cells. b.d., below detection. (**B**) RT-qPCR analyses of cytokine induction upon infection. MPI-2 cells were inoculated with different AdVs (350 cell-bound particles, 37°C, 30 min), washed, and incubated for the time indicated. Reverse-transcribed mRNA was amplified by qPCR, and results were expressed as fold change by the 2^−(ΔΔCt)^ method using hypoxanthine-guanine phosphoribosyltransferase cDNA as reference. Statistical significance from one-way ANOVA with Holm-Sidak correction for multiple comparisons. ***P < 0.001. (**C**) Verification of shRNA-mediated cGAS knockdown in MPI-2 cells. RNA from MPI-2, shcGAS, or shEGFP-treated cells was extracted, and cGAS mRNA levels were analyzed as described in (B). (**D**) cGAS promotes up-regulation of cytokine transcripts in virus-infected cells. MPI-2, shcGAS, or shEGFP cells were infected, processed, and analyzed as in (A and B). (**E**) cGAS promotes up-regulation of cytokine secretion from infected cells. Supernatant from MPI-2 control, shcGAS, or shEGFP treated AdV cells were quantified with a mouse CCL5 ELISA kit. Statistical significance as in (B).

The results so far showed that protein V secures the vDNA in the capsid in transit through the cytoplasm. We next tested whether protein V was involved in suppressing the induction of innate immune responses in the nontransformed, granulocyte-macrophage colony-stimulating factor (GM-CSF)–induced macrophage-like MPI-2 cell line (*41*). Cells were inoculated with wild-type and protein V–minus AdV particles such that similar numbers of particles were bound to cells (fig. S3A). mRNA levels of cytokines that were reported to be induced upon AdV infection (*42*) were then measured by reverse transcriptase quantitative polymerase chain reaction (RT-qPCR) at 5 and 10 hours pi. Notably, AdV-C5-∆V–infected cells secreted higher levels of type I IFNβ than wild-type AdV-C5, as measured by mouse embryonic fibroblast reporter cells expressing firefly luciferase under the control of the endogenous type I IFN inducible Mx2 promoter (MEF-Mx2-Luc-βKO) (Fig. 4A) (*41*). In agreement, interleukin-1α (Il1a), Ccl2, Cxcl2, and Ccl5 mRNAs were induced to higher levels by AdV-C5-∆V than wild-type AdV-C5 (Fig. 4B, displayed in blue and gray bars, respectively). These results were confirmed by single-cell analyses of Ccl2 mRNA using RNA fluorescence in situ hybridization (FISH) assay with branched DNA signal amplification (fig. S3, B and C). The induction of Ccl2 and Ccl5 was strongly attenuated by short hairpin RNA (shRNA)–mediated knockdown of the DNA sensor cGAS, an important signal transducer of mislocalized cytosolic DNA in infection and disease (Fig. 4, C and D, and fig. S3D), including AdV (*5*, *10*). The results were validated by CCL2 and CCL5 enzyme-linked immunosorbent assay (ELISA) showing that AdV-C5-ΔV increased the protein levels of these cytokines to much higher levels than the wild type, notably in a cGAS-dependent manner (Fig. 4E and fig. S3E). Together, these results indicate that the presence of protein V in AdV particles secures the viral genome in the capsid and thereby reduces the levels of DNA-PAMPs in the cytosol and restricts the innate response.

### Protein V dissociates from incoming virions and is ubiquitinated in a Mib1-dependent manner

**Fig. 5.**
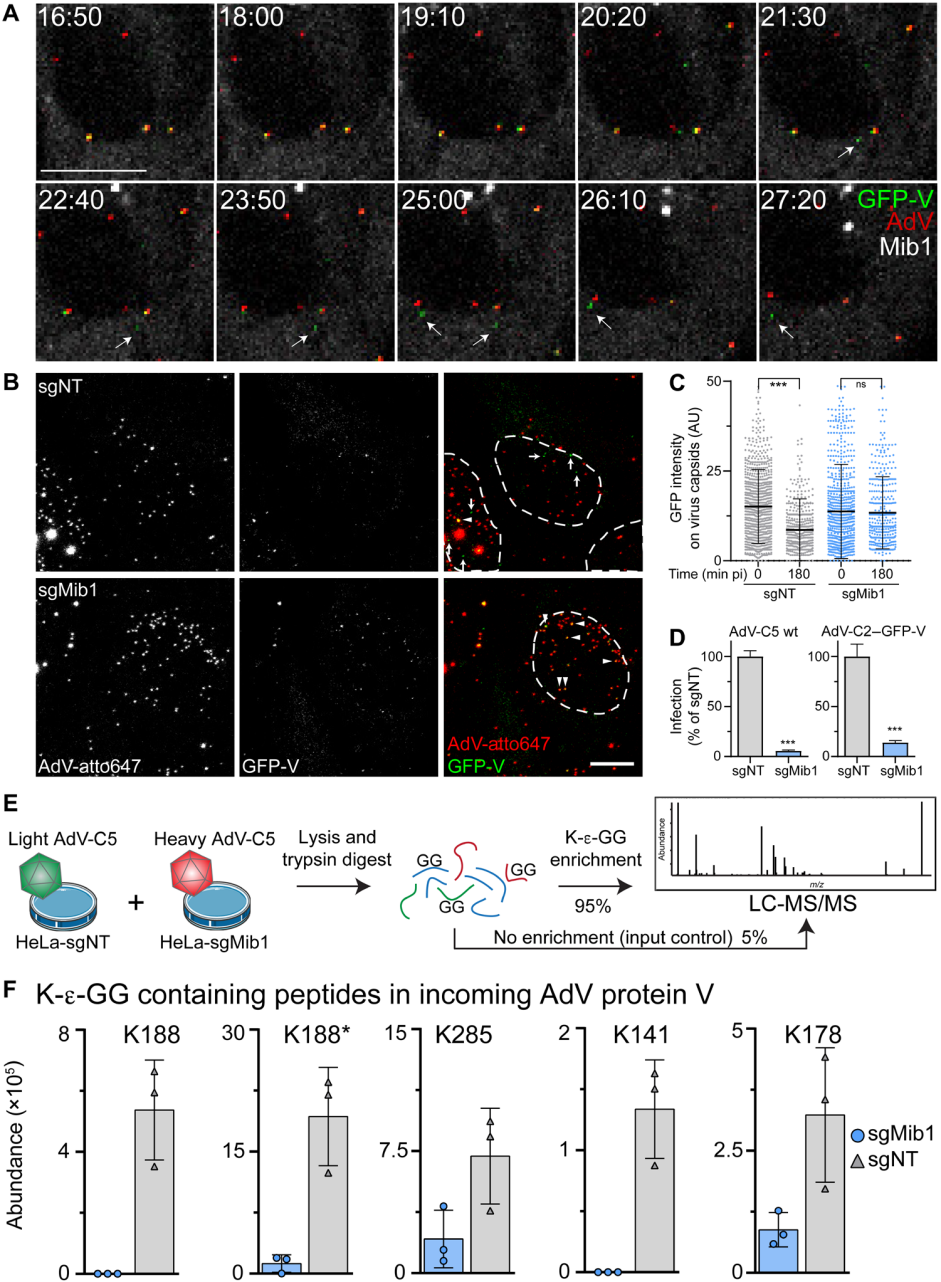
The core protein V dissociates from the virion at the NPC and is ubiquitinated during virus entry. (**A**) Montage of selected time points from movie S1. Time stamps are in minutes:seconds. Arrows indicate free GFP-V puncta after capsid dissociation. Three capsids containing GFP-V are visible at the nucleus, two of which discharge their GFP-V. Scale bar, 10 μm. (**B**) Mib1 promotes protein V release from AdV. HeLa-sgNT or HeLa-sgMib1 cells were inoculated with AdV-C2–GFP-V–ATTO647 (30 min), washed and incubated for 120 min, fixed, DAPI-stained (nuclear outlines), and imaged as described. Images are maximum projections. Arrows indicate free GFP-V; arrowheads indicate GFP-V–positive virus particles. Scale bar, 10 μm. (**C**) Time-dependent decrease of GFP-V on incoming virus capsids in control but not Mib1-KO cells. Cells were cold-bound with AdV-C2–GFP-V–ATTO647, washed, incubated at 37°C for 0 or 180 min, stained with DAPI, and imaged as described. Capsids were segmented on the basis of their ATTO647 signal. Each dot represents one virus particle. Data show means ± SD. Statistical significance was assessed as described in Fig. 1E. ***P < 0.001. AU, arbitrary units. (**D**) Mib1 is required for efficient AdV-C5 and AdV-C2–GFP-V infections. HeLa-sgNT and sgMib1 cells were infected at a multiplicity of infection (MOI) of 0.4, fixed after 24 hours, and stained with anti–protein VI antibody and DAPI. Nuclei were segmented, and infection was assessed on the basis of pVI signal. Data show means ± SD. *P* values from unpaired *t* tests. ***P < 0.001. (**E**) Workflow of di-Gly immunoprecipitation (IP) and analysis by LC-MS/MS following incubation of cells with light- or heavy-labeled AdV-C5. (**F**) Mib1 promotes ubiquitination of protein V on incoming virus particles. HeLa-sgNT cells and HeLa-sgMib1 cells were incubated for 2 hours with light- or heavy-labeled AdV-C5, lysed, anti–di-Gly immunoprecipitated, and analyzed by MS. Four ubiquitination sites on protein V were identified on five different peptides, as indicated by the GG-containing lysine. K188* denotes a peptide with a missed cleavage site. Results from three independent replicates are shown.

We next explored the dynamics of incoming protein V. ATTO647labeled AdV-C2–green fluorescent protein (GFP)–V particles bearing red ATTO647 fluorophores and green GFP-V fusion proteins readily reached the nucleus of mScarlet-Mib1 expressing Mib1-KO cells (HeLa-sgMib1) approximately 30 min pi. About 20 min after docking at the NPC, GFP-V puncta readily diffused away from the capsids without detectable translocation into the nucleus (Fig. 5A and movie S1), while vDNA ended up in the nucleus or got misdelivered to the cytosol (*17*, *35*). Notably, GFP-V puncta readily dissociated from capsids in HeLa cells treated with a non-targeting control guide RNA (HeLa-sgNT), but not HeLa-sgMib1 cells (Fig. 5, B and C). The lack of Mib1 strongly blocked AdV-C2–GFP-V infectivity, similar to AdV-C5-wt (Fig. 5D), indicating that GFP-V is an authentic reporter in virus entry, in agreement with an earlier report (*18*). The data show that the displacement of protein V from capsids at the nuclear envelope depends on Mib1 and occurs before vDNA import into the nucleus.

We next used a differential affinity purification MS strategy to determine whether incoming AdV particles were differentially ubiquitinated depending on Mib1 (Fig. 5E). First, we used SILAC (stable isotope labeling by amino acid) in cell culture to produce light and heavy AdV-C5 labeled with ^13^C_6_ lysine and ^13^C_6_^15^N_4_ arginine, respectively. Control HeLa-sgNT and sgMib1 cells were inoculated for 2 hours with equal amounts of light or heavy AdV-C5, respectively, lysed under strong denaturing conditions, and digested with trypsin protease. Trypsin cleaves the polypeptide chain C-terminal of lysine (K) and arginine (R) residues and leaves a di-glycine (Gly; G) remnant on the ε-amino group of the ubiquitinated K residue, a peptide motif that is specifically recognized by a monoclonal antibody (*43*). An aliquot of the digested lysate was not subjected to di-Gly enrichment. Using liquid chromatography–tandem MS (LC-MS/MS) with this sample, we identified peptides of the major capsid proteins hexon and penton base, the minor capsid proteins IIIa, VI, VIII, and IX, and the two core proteins V and VII from both heavy and light AdVs, indicating highly sensitive detection of incoming virus (table S1 and data S2). The di-Gly immunoprecipitates contained peptides from more than 4000 cellular proteins and three viral proteins: penton base, protein V, and VI (table S1 and fig. S4A). Except for protein VI (*25*), virion proteins were not previously described to be ubiquitinated. Ubiquitinated peptides from penton base and protein VI were present in similar amounts in both sgNT and sgMib1 lysates, whereas ubiquitinated peptides from protein V were strongly enriched in sgNT cells, specifically the residues K141, K178, K188, and K285 (Fig. 5F; fig. S4, B and C; and data S2). Notably, before di-Gly enrichment, the viral proteins from the heavy and light AdV inocula were similar in abundance (fig. S4C and data S2), indicating that Mib1 promotes the ubiquitination of incoming protein V. However, MS and shotgun proteomics of purified AdV-C5 gave no evidence for ubiquitination of protein V residues (fig. S4D).

### Protein V ubiquitination enhances vDNA nuclear import and infection

**Fig. 6.**
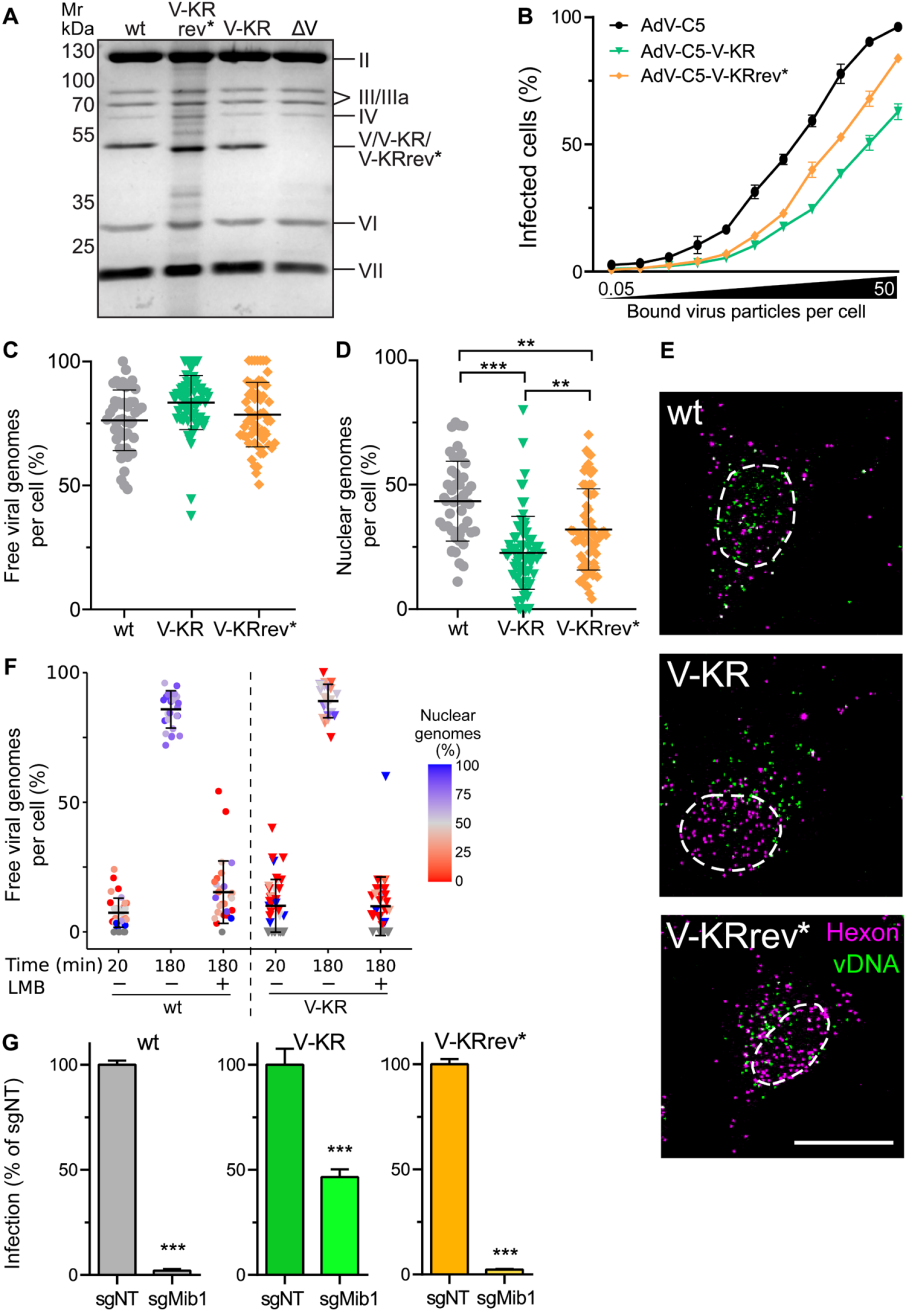
Protein V mutagenesis reveals that K178 and K188 are sufficient for incoming AdV particles to respond to Mib1 uncoating cues. (**A**) Protein V abundance in purified AdV-C5, AdV-C5-V-KRrev*, and AdV-C5-V-KR particles. Equal amounts of virion extracts, including AdV-C5-ΔV, were separated by SDS-PAGE and stained using QuickBlue. (**B**) MOI-resolved AdV-C5, AdV-C5-V-KR, and AdV-C5-V-KRrev* infections. HeLa cells were infected with virus (0.05 to 50 particles cold-bound per cell), washed, incubated (37°C, 24 hours), fixed, and stained with anti-E1A and DAPI. Infection was scored by percentage of E1A-positive nuclei. Data show means ± SD. (**C** to **E**) Uncoating of incoming AdV genomes. HeLa cells were incubated with genome-labeled AdV (30 min); washed; chased for 180 min; fixed; and stained for hexon, nuclei (DAPI, nuclear outlines), and vDNA. Data show means ± SD. Statistical significance was assessed as described in Fig. 1E. Representative images from maximum projections. Scale bar, 10 μm. **P < 0.01 and ***P < 0.001. (**F**) Stable AdV-C5-V-KR particles in the cytoplasm. HeLa cells were incubated with genome-labeled AdV-C5 or AdV-C5-V-KR with/without LMB (50 nM), fixed, and stained with anti-hexon antibodies and click chemistry. Data show means ± SD. Color-coded percentage of capsid-free genomes over the nucleus. Statistical significance assessed as in (D). (**G**) Ubiquitination of protein V K178 and K188 restores infection dependence on Mib1. HeLa-sgNT and sgMib1 cells were infected for 24 hours (MOI, 0.4), fixed, and stained with anti–protein VI and DAPI (nuclei), and classified on the basis of their VI signal. Data show means ± SD. *P* values from unpaired *t* tests. ***P < 0.001.

To address the functional significance of protein V ubiquitination, we abrogated the canonical ubiquitination sites of protein V by mutating all the 26 lysine residues to arginine and engineered a new virus, AdV-C5-V-KR. As a control, we reverted two arginine residues to lysine at positions 178 and 188 and generated the so-called AdV-C5-V-KRrev*. K178/188 were ubiquitinated in wild-type but poorly in Mib1-KO cells (see Fig. 5F). Purified AdV-C5-V-KR and AdV-C5-V-KRrev* incorporated protein V at amounts comparable to the parental AdV-C5, as shown by SDS-PAGE and QuickBlue protein staining (Fig. 6A). While protein V-KR and V-KRrev* migrated slightly faster than wild-type protein V, there was no difference in any of the other capsid proteins. The infectivity of AdV-C5-V-KR particles was strongly impaired compared to the parental AdV-C5 (Fig. 6B), very similar to the AdV-C5-ΔV mutant (see Fig. 1D), while AdV-C5-V-KRrev* infectivity was increased, albeit not to wild-type levels. Although both the AdV-C5-V-KR and the AdV-C5-V-KRrev* particles showed high overall vDNA release from capsids like wild type, the nuclear import efficiency of V-KR vDNA was only 25% of the incoming genomes, about half of wild-type AdV-C5 (Fig. 6, C to E). Nuclear import of AdV-C5-V-KRrev* was enhanced although not quite to wild-type levels. The data argue that ubiquitination of V at residues K178/K188 enhances vDNA nuclear import. Non-ubiquitinable protein V bearing particles were as stable as wild-type AdV-C5 in LMB-treated cells, where virus docking to the NPC is precluded, indicating that ubiquitination of protein V was not required for securing the capsid vDNA during cytoplasmic transport (Fig. 6F).

To test whether ubiquitination of K178 and K188 was sufficient to restore the infection dependence on Mib1, we used AdV-C5-V-KRrev* to infect Mib1-KO cells. The reverted AdV-C5-V-KRrev* was strongly dependent on Mib1, similar to AdV-C5, indicating that K178/188 ubiquitination required Mib1 and was crucial for infection (Fig. 6G). The infection of Mib1-KO cells with AdV-C5-V-KR was more efficient than wild-type AdV-C5, but not as efficient as AdV-C5-∆V, suggesting that Mib1 is involved in ubiquitination of an additional target besides protein V to gate nuclear import of the vDNA from capsids containing protein V. Together, the results show that the vDNA core–associated protein V serves as a linchpin securing the stability of cytoplasmic AdV and becomes a Mib1-dependent ubiquitin-responsive target at the NPC facilitating the nuclear import of vDNA upon virion rupture.

### The proteasome activity is required for capsid disassembly

**Fig. 7.**
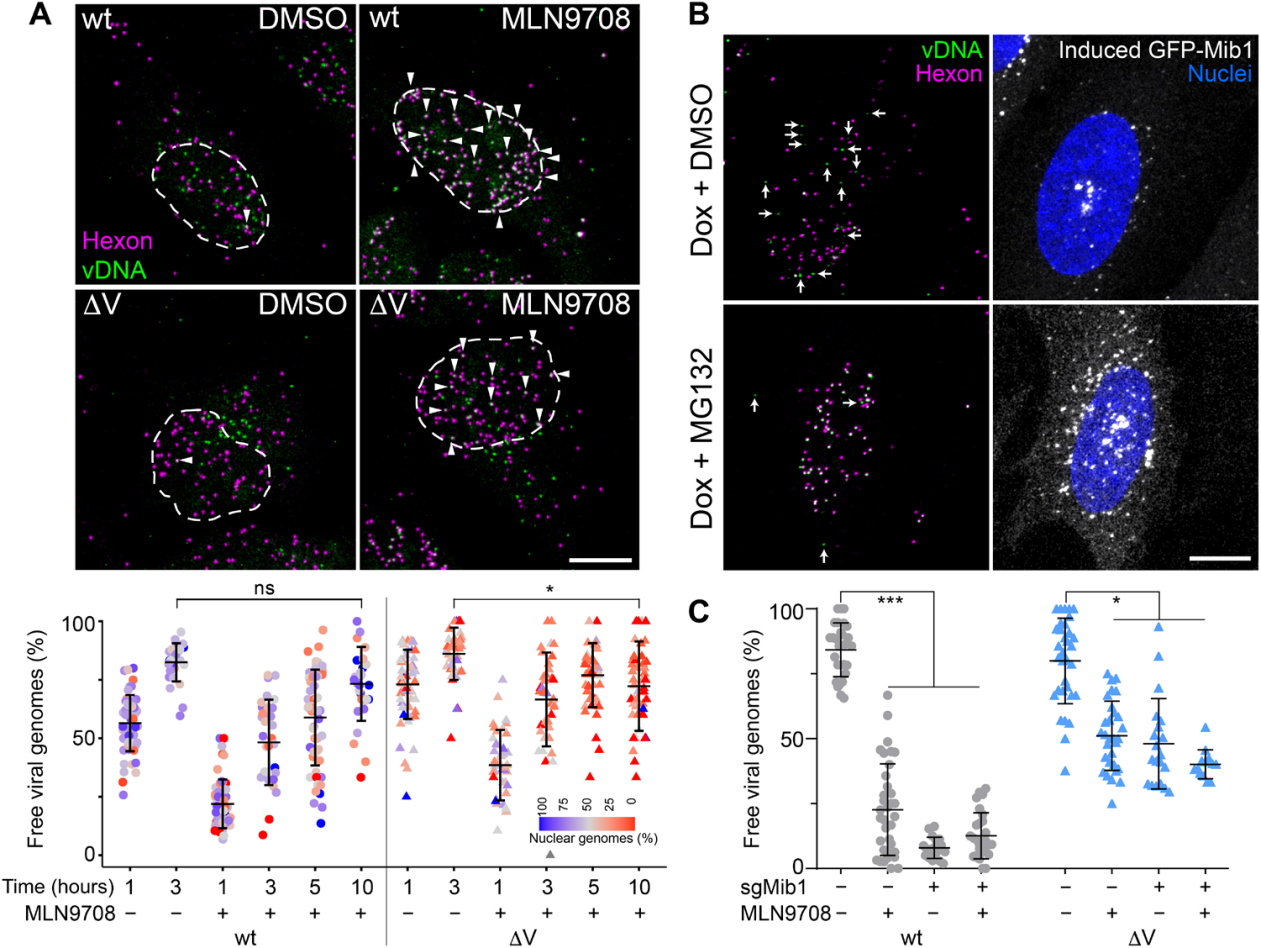
Proteasome activity required for capsid disassembly at the NPC. (**A**) The proteasome enhances uncoating of virus genomes independently of protein V. HeLa cells were incubated with genome-labeled AdV-C5 or AdV-C5-ΔV as indicated. MLN9708 (10 μM) was present during the entire experiment. Data are represented as maximum projection images 180 min pi. Arrowheads indicate viral particles containing vDNA. Viral genomes were segmented and classified as capsid-free on the basis of their hexon intensity. Ratio of the number of capsid-free genomes over the nuclear mask is color coded. Statistical significance was assessed as described in Fig. 1E. ***P < 0.001 and *P < 0.05. Scale bar, 10 μm. DMSO, dimethyl sulfoxide. (**B**) Proteasome activity promotes uncoating of viruses docked at the NPC. HeLa-sgMib1 cells carrying a tetracycline-inducible GFP-Mib1 cassette were incubated for 1 hour with wild-type AdV-C5-EdC; washed; incubated at 37°C for 2 hours; treated with doxycycline (1 μg/ml) or MG132 (10 μM) for 5 hours; fixed; stained for vDNA, hexon, and nuclei; and imaged by confocal fluorescence microscopy. Images are maximum projections. Arrows indicate free viral genomes. Scale bar, 10 μm. (**C**) Proteasome-mediated enhancement of vDNA/capsid separation does not require Mib1. HeLa-sgNT and sgMib1 cells were incubated with genome-labeled AdV-C5 or AdV-C5-ΔV as described in (A). MLN9708 (10 μM) was present during the entire experiment. Cells were fixed 180 min pi and stained as described in (A). Viral genomes were segmented and classified as capsid-free on the basis of their hexon intensity. Statistical significance was assessed as described in Fig. 1E. *P < 0.05 and ***P < 0.001. All the other differences in pairwise comparisons within AdV-C5 and AdV-C5-ΔV, respectively, are not significant.

To address whether additional ubiquitination targets besides protein V are involved in capsid disassembly and vDNA nuclear import, we investigated the effect of proteasomal inhibition with the chemical compounds MLN9708 and MG132. Both inhibitors strongly reduced AdV-C5 infection of HeLa cells measured by late viral protein expression 24 hours pi, with inhibitory concentrations of 50% efficacy (IC_50_) of 0.082 and 0.176 μM for MLN9708 and MG132, respectively (fig. S5, A and B). Very similar results were obtained with human diploid fibroblasts immortalized with telomerase (fig. S5C). MLN9708 washout experiments at 2 hours pi showed strong inhibition of infection of AdV-C5, AdV-C5-ΔV, and AdV-C5-V-KR infections, indicating that all three viruses require an active proteasome for entry (fig. S5D). Using confocal fluorescence microscopy, we tracked the incoming genomes of EdC-labeled AdV-C5 and AdV-C5-ΔV. MLN9708 reduced the number of vDNA released from wild-type AdV-C5 and AdV-C5-ΔV and lowered the amount of nuclear vDNA up to 3 hours pi, showing that the proteasome enhances vDNA uncoating and infection independent of protein V (Fig. 7A). This was distinct from the proteasome-independent premature dismantling of AdV-C5-ΔV in the cytoplasm, as shown in the presence of LMB (fig. S2C). To further test whether the proteasome acted upstream of DNA release at the NPC, we allowed AdV-C5 to reach the nucleus in Mib1-KO cells for 3 hours and then induced the expression of GFP-tagged Mib1 with doxycycline. In the absence of proteasomal inhibitors, vDNA was readily released from the capsids, whereas the presence of MG132 strongly reduced the levels of capsid-free vDNA (Fig. 7B), indicating that proteasomal activity is involved in the release of vDNA from capsids docked at the NPC.

In the absence of Mib1, proteasome inhibition by MLN9708 did not show any further reduction of DNA release from AdV-C5-wt, most likely because of technical limitations in measuring additional inhibitory effects on top of the strong infection block in Mib1-KO cells (Fig. 7C). Note, however, that the inhibitory effect of MLN9708 on nuclear import of vDNA in wild-type infection was transient and no longer observed at 10 hours pi, suggesting that the proteasome has a kinetic role in enhancing vDNA nuclear import (Fig. 7A). On the other hand, genome release from AdV-C5-ΔV virions, which prematurely uncoat in the cytoplasm independent of Mib1, was hardly inhibited by MLN9708 (or in Mib1-KO cells), indicating that the premature release of the genome is independent of the proteasome, although the decomposition of the dismantled capsid may still depend on the proteasome. Collectively, the data show that the proteasome supports AdV-C5 DNA uncoating by targeting protein V in a Mib1-dependent manner and involves another target yet to be identified.

## DISCUSSION

**Fig. 8.**
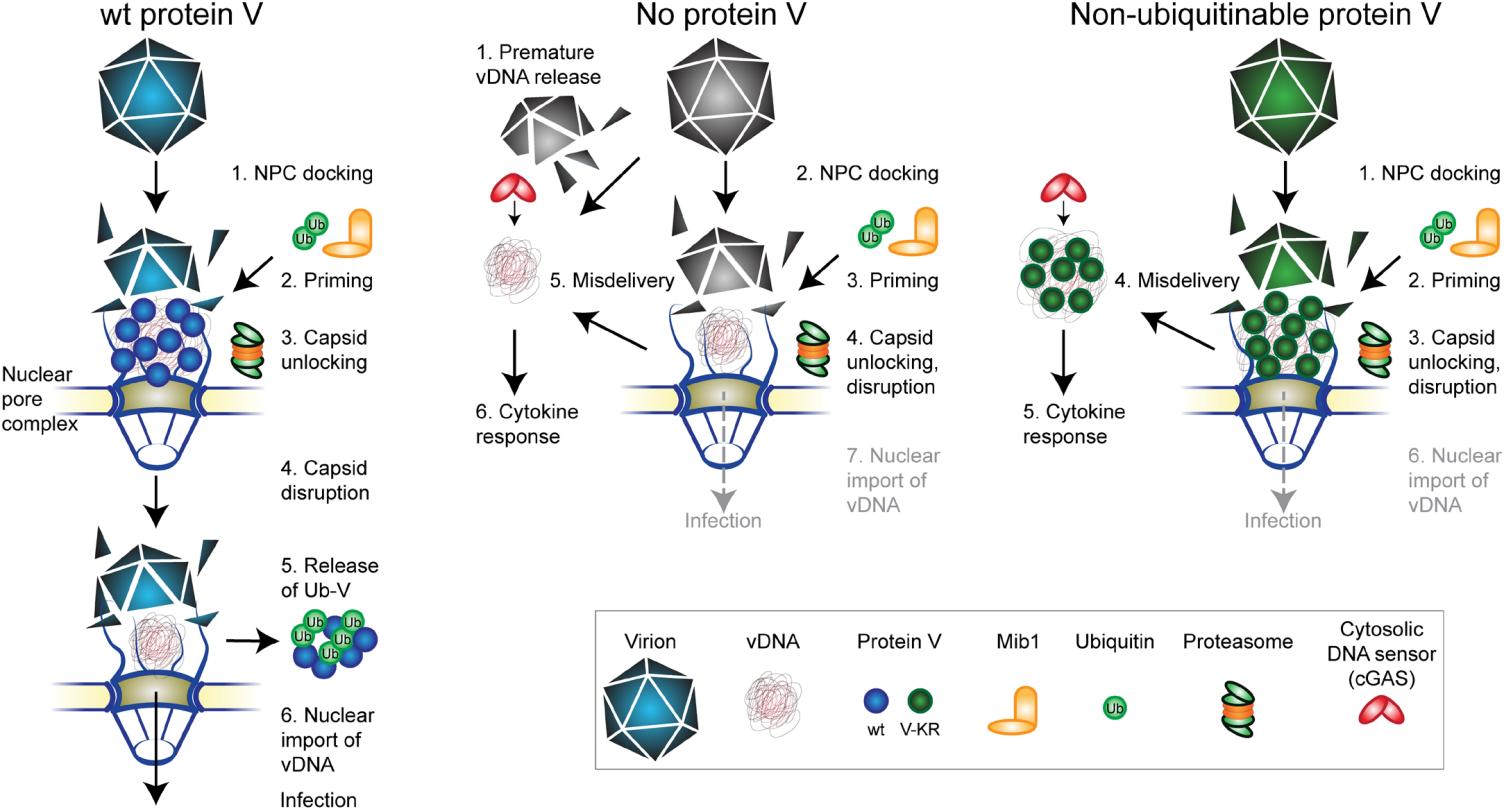
Schematic model depicting how the linchpin protein V enhances cytoplasmic AdV stability and ubiquitination by Mib1 triggers vDNA delivery into the nucleus. Upon docking at the NPC, Mib1-mediated ubiquitination primes the AdV-wt capsid for vDNA release and nuclear import (**left**). Protein V ubiquitination triggers V release from the capsid and, together with the proteasome activity, facilitates the import of the viral genome into the nucleus. Non-ubiquitinable protein V fails to separate from the vDNA and prevents vDNA nuclear import (**right**). Absence of protein V reduces the stability of the virions in the cytoplasm, which leads to the premature release of vDNA in the cytoplasm, distant from the nucleus (**center**). Free cytoplasmic genomes activate the DNA sensor cGAS, triggering a strong cytokine response.

The work here identifies a dual function for the internal virion protein V. Protein V serves as a linchpin securing the vDNA in the capsid and tunes virion dismantling at the NPC and vDNA import into the nucleus in a proteasome-dependent process. The data are based on two novel engineered AdVs, one lacking protein V without compensatory genomic mutations and one containing non-ubiquitinable protein, as well as cells lacking the E3 ubiquitin ligase Mib1 (Fig. 8). Mib1-KO cells are resistant to AdV-C5 infection (*35*) but are susceptible to AdV lacking protein V or bearing non-ubiquitinable protein V, although the latter are less infectious than wild-type virus. The genomes of protein V mutant AdVs were poorly imported into the nucleus and uncoated in the cytosol, thereby enhancing chemokine and IFN production through the DNA sensor cGAS.

Viruses typically evade from the cGAS/signaling protein, stimulator of IFN genes (STING) pathway by two principal mechanisms, inhibition of signal transduction, and limitation of genome exposure, as shown with herpes simplex virus 1, which reduces the levels and catalytic activity of cGAS (*44*), and HIV using its capsid to shield the genome from the cGAS/STING pathway, respectively (*45*). We show that the AdV protein V suppresses the cGAS/STING pathway activation by securing the vDNA in the capsid. Protein V thereby complements the immediate early AdV protein E1A, which binds to STING and suppresses IFN signaling (*4*). cGAS/STING is key in DNA sensing and integrates a range of signals from both DNA and RNA viruses, which mislocalize double-stranded viral or cellular DNA to the cytosol (*44*). cGAS activation raises 2′ to 5′ and 3′ to 5′–cyclic GMP-AMP second messengers (cGAMP), which bind to STING. Activated STING induces type I IFNs through TANK (TRAF-associated NF-κB activator) binding kinase 1 and IFN regulatory factor 3 (IRF3), as well as IL-6 and tumor necrosis factor–α through the transcription factor nuclear factor κB (*46*).

Protein V is present in mastadenoviruses infecting mammals but not in AdVs infecting lower vertebrates (*47*). Notably, in lower metazoans, the C-terminal tail of STING lacks the mammalian motif to recruit IRF3 and activate type I IFNs (*48*). It is possible that mastadenoviruses evolved protein V to keep the cGAS/STING pathway in check and suppress the transmission of alarm signals, such as cGAMP. Protein V potentially also limits the establishment of an antiviral state in neighboring cells and favors viral spreading. The activation of cGAS requires a critical threshold of the mislocalized cytoplasmic double-stranded DNA and involves liquid-phase condensation (*49*). It is unlikely that nuclear AdV DNA activates cGAS even if the sensor translocates to the nucleus. The vDNA delivered into the nucleus moves slowly and does not aggregate (*39*), unlike cytoplasmic vDNA, which is mobile and can aggregate (*17*). Accordingly, the incoming protein V restricting cGAS/STING exclusively localizes to the cytoplasm.

AdVs devoid of protein V were less stable than wild type and prematurely dismantled in the cytoplasm, possibly because protein V present in 160 copies normally acts as a capsid glue by connecting the vDNA and the capsid wall (*50*, *51*). In the virion, protein V balances the negative charges of the vDNA together with proteins VII, X/μ, and IVa2, akin to a histone H1–like protein. The process, which prematurely dismantles protein V–minus AdV is likely distinct from the one, which inactivates antibody-coated virus particles, as the latter involves the proteasome (*52*). AdVs loaded with antibodies become subject to inactivation through recognition of the immunoglobulins by the E3 ubiquitin ligase TRIM21 (tripartite motif 21), whereas the premature dismantling of the protein V–minus virions depended, at least in part, on Mib1. The molecular mechanisms leading to premature uncoating of protein V–minus AdV in the cytoplasm remain to be characterized, but pulling forces from microtubule-associated motors do not seem to be involved.

In contrast, the AdV-C5-wt particles resisted not only the cytoplasmic crowding but also the pulling forces from dynein and kinesin motors during cytoplasmic transport, allowing the particles to reach the NPC (*53*, *54*). NPCs selectively gate transport for macromolecules as large as ribosomal subunits and small virus particles into and out of the nucleus (*3*, *55*). The cytoplasmic periphery of the NPC is made up of Nup214, Nup88, and Nup358, which form flexible ~36-nm-long filaments (*56*). Nup214 and Nup358 function as the AdV docking site and the recruiter of the uncoating motor kinesin-1, respectively (*19*). Here, we showed that the efficiency of nuclear translocation of the AdV genome depended on the E3 ligase Mib1 and ubiquitination of protein V. Ubiquitination analyses by affinity purification MS identified four lysine residues on protein V that carried a di-Gly remnant after tryptic digest. Di-Gly remnants on the ε-amino group of lysines derive from digestion of proteins conjugated to ubiquitin or ubiquitin-like modifiers, such as ISG15 (interferon-stimulated gene15) and NEDD8 (neuronal precursor cell-expressed developmentally down-regulated protein 8), which cannot be distinguished from ubiquitin by MS (*43*). It is unlikely that the di-Gly remnants on protein V were derived from another modification than ubiquitin since our detection methodology was reported highly selective for ubiquitination (>94% of all the K-ε-di-Gly sites) and only 6% for NEDD8ylation or ISG15ylation (*57*). Notably, purified AdV-C5 gave no sign of protein V ubiquitination, in line with LC-MS data of purified AdV-C2 particles (*58*), indicating that protein V ubiquitination happened during viral entry rather than progeny formation or egress.

Protein V potentially provides a direct ubiquitination substrate for Mib1. Mib1-KO cells allowed full infection with AdV particles lacking protein V, which emphasizes the importance of Mib1-dependent ubiquitination of protein V in releasing viral genomes from stabilized capsids and nuclear import. Proteasomal inhibitors blocked genome import independently of protein V, suggesting the presence of a factor that is subject to proteasomal degradation for viral uncoating at the NPC. We speculate that this factor is a cellular protein that stabilizes the viral capsid against disruption by kinesin-mediated pulling forces, which had been implicated in capsid disruption at the NPC (*19*). It is plausible that ubiquitination and proteasomal degradation of this factor occurs at the NPC since the proteasome inhibitor MG132 prevented the vDNA release from the NPC-docked capsid upon induction of Mib1 expression in Mib1-KO cells. A recent study identified an increase in ribonucleoproteins (RNPs) as putative Mib1 substrates upon incubation of the cells with AdV (*36*). It is conceivable that an RNP in the vicinity of the nucleus interacts with protein V or the AdV capsid to facilitate the disassembly of the capsid before removal of protein V from the vDNA and the translocation of the genome through the NPC.

The role of the proteasome in AdV capsid disassembly at the NPC represents a novel function and can now be studied in more detail. It extends the role of the proteasome beyond binding, internalization, and trafficking of other viruses (*59*) and increases the therapeutic potential of proteasome inhibitors. Developing proteasomal inhibitors against vDNA uncoating may also be beneficial in reducing cytokine response and inflammation. Genetic perturbations of protein V in AdV-C5-ΔV, AdV-C5-V-KR, and AdV-C5-V-KRrev* increased the levels of viral genomes in the cytosol as a result of unscheduled vDNA release or misdelivery. The AdV-C5-ΔV particles led to the largest overall induction of innate cytokines, strongly dependent on the presence of the cGAS sensor. We speculate that the non-ubiquitinable V-KR protein partially shields misdelivered vDNA from cellular sensors. Since protein V occurs in no other AdV genus than *Mastadenovirus* where it is highly conserved (*60*, *61*), we speculate that the viruses lacking protein V evolved other mechanisms to secure their capsid DNA before arriving at the nucleus. Misdelivery of vDNA is, however, not unique to AdV. For example, more than half of HIV-1 reverse-transcribed genomes were found to be capsid-free in the cytosol of primary human macrophages (*62*). It remains to be explored how other viruses secure and shield their vDNA from cellular sensors or activate cytosolic DNA sensors (*63*). In summary, the mechanisms uncovered here advance the understanding of how viruses control the exposure of their genomes, a crucial process in pathology and the development of customized gene delivery vehicles and vaccines for antiviral and cancer therapy and oncolytic viruses.

## MATERIALS AND METHODS

### Cell culture and virus production

HeLa-ATCC, A549, HDF-TERT, human embryonic kidney 293T, and HER911 cells were maintained in Dulbecco’s modified Eagle’s medium (DMEM; Gibco) supplemented with non-essential amino acids (Thermo Fisher Scientific) and 10% fetal calf serum (FCS; Gibco). During infection experiments, the medium was additionally supplemented with penicillin (100 U/ml) and streptomycin (100 μg/ml). The cells were grown at 37°C in a 5% CO_2_ atmosphere for no longer than 20 passages. HeLa-sgNT, HeLa-sgMib1, and HeLa-sgMib1 cells carrying a tetracycline-inducible GFP-Mib1 cassette have been previously described (*35*). HeLa-sgMib1 cells expressing mScarlet-Mib1 were generated by lentiviral transduction followed by selection with puromycin (2 μg/ml). MPI-2 cells were maintained in RPMI 1640 medium (Sigma-Aldrich) supplemented with 10% FCS and GM-CSF (10 ng/ml; Miltenyi Biotec) (*41*, *64*). MEF-Mx2-Luc-βKO cells have been previously described (*41*).

All AdVs were grown in A549 cells and purified over two cesium chloride gradients as previously described (*65*). AdV-C5 (wt300) has been previously described (*66*). AdV-C5-ΔIX was provided by R. Hoeben (Leiden University Medical Center, The Netherlands) (*67*). AdV-C2–GFP-V was used as described (*18*). Capsid-labeled viruses were generated as described (*68*). Genome-labeled AdV was produced by growing the virus in A549 cells in the presence of 2.5 μM EdC (Jena Biosciences) (*17*). AdV-C5-ΔV, AdV-C5-V-KR, and AdV-C5-V-KRrev* were generated by recombineering from the pKSB2 bacmid, which contains the entire AdV-C5 wt300 genome (*66*, *69*). Heavy AdV-C5 carrying ^13^C_6_ lysine and ^13^C_6_^15^N_4_ arginine was produced in HeLa cells that were grown in DMEM supplemented with ^13^C_6_ lysine and ^13^C_6_^15^N_4_ arginine (Thermo Fisher Scientific).

### Generation of AdV mutants

In the first step, the galactokinase (GalK) cassette was amplified by PCR from the pGalK plasmid using primers that carried 45–base pair homology sequences directly up- or downstream of the protein V coding region (see table S2, primers GalK_f and GalK_r). The PCR product was purified by gel extraction and digested with Dpn I (Promega) for 1 hour to remove residual template DNA before a second round of purification. Electrocompetent *Escherichia* *coli* SW102 cells harboring the AdV-C5 containing bacmid (pKSB2) were then electroporated with the purified PCR construct. Positive clones were verified by sequencing and underwent a second electroporation reaction. In the case of AdV-C5-ΔV, this was done with a dsDNA oligonucleotide (dV_f and dV_r; table S2) consisting of the left and right homology sequences. For the AdV-C5-V-KR mutant, the recombination substrate was a synthesized modified protein V DNA sequence in which all lysine codons were replaced by arginine codons flanked by the left and right homology arms (table S2; synthesized by Thermo Fisher Scientific). The resulting AdV-C5-V-KR bacmid then served as the template for constructing the AdV-C5-V-KRrev* mutant. Bacmid DNA from positive clones was extracted, digested with Pac I to release the AdV genome, and transfected into HER911 cells. Rescued viruses were plaque-purified, expanded, and verified by sequencing.

### Single-round infection assays

Ten thousand cells were seeded in a black 96-well imaging plate. On the following day, the virus was diluted in infection medium (DMEM supplemented with 2% FCS, non-essential amino acids, and penicillin/streptomycin) to reach an infection of about 40%. The culture supernatant was aspirated, and 100 μl of diluted virus suspension was added to the cells. The cells were fixed with 4% paraformaldehyde (PFA) in phosphate-buffered saline (PBS) for 10 to 15 min at room temperature. The remaining PFA was quenched with 25 mM NH_4_Cl diluted in PBS for 5 to 10 min, followed by permeabilization with 0.5% Triton X-100 in PBS for 3 to 5 min. Cells were stained with rabbit anti–protein VI (*68*) or mouse anti-E1A clone M73 (Millipore, 05-599) diluted in blocking buffer (10% goat serum in PBS) for 1 hour at 4°C. After three washes of 4 min each in PBS, cells were stained with secondary antibody (goat anti-rabbit Alexa Fluor 488 or goat anti-mouse Alexa Fluor 488, Thermo Fisher Scientific) diluted in blocking buffer containing 4′,6-diamidino-2-phenylindole (DAPI; 1 μg/ml) for 30 min at room temperature. After three more washes of 4 min in PBS, cells were imaged in a Molecular Devices high-throughput microscope (IXM-XL or IXMc) in wide-field mode with a 20× objective. For quantification of infection with CellProfiler (*70*), nuclei were segmented according to the DAPI signal, and the intensity of the infection marker over the nuclear mask was measured.

### Virus particle infectivity

Virus input was normalized to the number of viral particles that bound to the cells, which was determined in a binding assay. To this end, HeLa cells grown on coverslips in a 24-well plate were incubated with virus for 1 hour on ice, after which the virus inoculum was washed away and cells were immediately fixed with 4% PFA. Bound viral particles were stained with mouse anti-hexon 9C12 antibody (*71*) and goat anti-mouse Alexa Fluor 488. Nuclei were stained with DAPI and cell outlines with Alexa Fluor 647–conjugated succinimidyl ester (Thermo Fisher Scientific). Samples were imaged using a Leica SP8 confocal laser scanning microscope (cLSM). Three-dimensional stacks were recorded, and the number of particles that bound to cells was quantified in maximum projections using CellProfiler. HeLa cells in a 96-well plate were then incubated with a 1:2 dilution series of virus starting from 50 bound particles per cell. After 1 hour on ice, virus inoculum was removed, and fresh medium was added. Cells were fixed at 20 hours pi and stained for E1A as described above. Multiplicity of infection (MOI) for infection assays was based on infectious particles in the particular assays. For example, MOI of 0.5 indicated that 50% of the cells were infected at the time of fixation.

### SDS-PAGE and Western blotting

Purified virus particles were lysed in SDS-PAGE lysis buffer [200 mM tris (pH 6.8), 10% glycerol, 5 mM EDTA, 0.02% bromophenol blue, 5% SDS, and 50 mM dithiothreitol (DTT)] and boiled for 5 min at 95°C. Samples were then loaded onto a 10% SDS-PAGE gel and transferred to a polyvinylidene difluoride membrane (Amersham). After blocking with blocking solution [5% milk powder in 20 mM tris, 150 mM NaCl, and 0.1% Tween 20 (pH 7.5)], the membrane was incubated with primary and secondary antibodies diluted in blocking solution at 4°C overnight or 1 hour at room temperature with four washes of TBST in between. Horseradish peroxidase (HRP)–coupled secondary antibody was detected using the ECL reagent (GE Healthcare). Primary antibodies used in Western blotting were rabbit polyclonal anti-hexon and rabbit polyclonal anti–protein V (both gifts from U. Pettersson). Secondary antibody used in Western blotting was goat anti-rabbit–HRP (Cell Signaling Technology, 7074). Chemiluminescence signals were detected using an ImageQuant LAS 4000 system. Alternatively, after gel electrophoresis, separated proteins were stained in the gel using Coomassie or QuickBlue protein stain (LubioScience).

### Click chemistry and vDNA analysis

Cells grown on coverslips were infected with genome-labeled AdV for various time points. Where specified, cells were incubated with specific inhibitors (50 nM LMB, 5 μM DBeQ, 10 μM MLN9708, and 10 μM nocodazole) 1 hour before, during, and after virus inoculation at 37°C. For microtubule disruption, cells were incubated in addition for 1 hour on ice in the presence of 10 μM nocodazole 1 hour before virus inoculation. After fixation, quenching, and permeabilization, samples were stained for incoming capsids with the 9C12 anti-hexon antibody. After primary and secondary antibody incubation, the coverslips were inverted onto a 30-μl droplet of click reaction mix for 2 hours at room temperature. The freshly prepared click reaction mix consisted of 10 μM Alexa Fluor 488–conjugated azide (Thermo Fisher Scientific), 1 mM CuSO_4_, and 10 mM sodium ascorbate in the presence of 1 mM tris-hydroxypropyltriazolylmethylamine (THPTA) (Sigma-Aldrich) and 10 mM aminoguanidine (Sigma-Aldrich) in PBS. Samples were stained with DAPI and Alexa Fluor 647–conjugated succinimidyl ester and imaged with a Leica SP8 cLSM as described above. Nuclei and single viral genomes and/or capsids were segmented according to the corresponding signal using CellProfiler. Genomes were classified as capsid positive on the basis of their corresponding hexon signal. Nuclear genomes were those that overlapped with the nuclear mask that was created on the basis of the DAPI signal. Percentage of nuclear genomes was set in relation to all capsid-free genomes.

### Confocal microscopy

A Leica SP8 cLSM was used in all experiments, in which single viral particles and genomes were imaged. Imaging was performed with a 63× magnification oil objective with a numerical aperture of 1.40 and a zoom factor of 2, with a pixel size of 0.181 μm. *Z* stacks were captured with a step size of 0.5 μm to capture the entire cell, and the size of the pinhole was 1 Airy unit. Leica hybrid detectors were used for each channel.

### Confocal spinning-disk live microscopy

Eight thousand HeLa-sgMib1 cells expressing mScarlet-Mib1 were seeded in a 10-well CELLview slide (Greiner Bio-One) with a 175-μm-thick cover glass embedded on its bottom. After 2 days, the cells were incubated with AdV-C2–GFP-V–ATTO647 (*18*) at 37°C for 30 min. Unbound virus was washed away; fresh medium without phenol-red was added to the cells, and live imaging was started on a Visitron CSU-W1 spinning disk microscope consisting of a Nikon Eclipse T1 microscope and a Yokogawa confocal scanning unit W1 with a stage top incubation system at 37°C and 5% CO_2_. Z stacks consisting of four steps with a step size of 1.4 μm were acquired every 10 s for up to 30 min with a 100× oil objective (numerical aperture 1.4) and a pinhole of 50 μm. The focus was maintained with a perfect focus system.

### Di-Gly immunoprecipitation and MS analysis

Di-Gly immunoprecipitation (IP) and MS analysis were performed essentially as described (*72*). HeLa-sgNT and sgMib1 cells were seeded in three 15-cm dishes each to a confluency of ca. 90%. Cells were incubated with 160 μg of heavy or light AdV-C5 in the presence of 10 μM MG132 (Sigma-Aldrich) for 2 hours at 37°C. After washing with PBS and trypsinization, cells were pelleted by centrifugation at 500*g* at 4°C for 4 min, resuspended in PBS, and centrifuged again. The cell pellet was resuspended in 1.3 ml of ice-cold lysis buffer comprising 8 M urea, 150 mM NaCl, 1 mM EDTA, 50 mM tris-HCl (pH 8), and 0.05 mM PR-619 protease inhibitor cocktail (Roche), followed by three cycles of sonication using a Hielscher sonicator for 1 min and chilling on ice for 1 min. Samples were then centrifuged at 16,100*g* at 4°C for 15 min, and the supernatant was collected. Protein concentration was determined via Micro BCA assay (Thermo Fisher Scientific). Five milligrams of each lysate was then mixed into one tube, followed by reduction and alkylation with 10 μM tris(2-carboxyethyl)phosphine (TCEP) and 40 μM chloroacetamide at room temperature in the dark for 30 min. The lysate was subsequently diluted to a concentration of 4 M urea by adding 50 mM tris (pH 8) and digested with 60 μg of LysC (Wako Chem) at 37°C under agitation for 4 hours. The lysate was then diluted to <2 M urea and incubated with 100 μg of trypsin (Promega) overnight at 37°C under agitation. The following day, 1% trifluoroacetic acid (TFA) was added to the sample followed by incubation on ice for 15 min. After centrifugation at 3000*g* for 10 min, the supernatant was transferred to a new tube and desalted using a 500-mg tC18 Sep-Pak cartridge (Waters) with a vacuum manifold according to the manufacturer’s instructions. Peptides were eluted with a 60% acetonitrile (ACN)/0.1% TFA solution and dried in a SpeedVac vacuum concentrator (Thermo Fisher Scientific). For the IP of di-Gly peptides, samples were incubated with cross-linked agarose–coupled PTMScan Ubiquitin Remnant Motif antibody (Cell Signaling Technology) according to the manufacturer’s instructions. Before the IP, a 5% aliquot was set aside for global proteomic analysis to estimate the amounts of viral proteins in the samples. Following elution with 0.15% TFA, samples were desalted using StageTips with two disks of C18 and elution with 60% ACN/0.1% TFA. Eluted samples were lyophilized in a SpeedVac and stored at −20°C until MS analysis. Samples were reconstituted in 3% ACN/0.1% formic acid (FA) for analysis by LC-MS/MS on an Orbitrap Q Exactive HF mass spectrometer (Thermo Fisher Scientific) coupled to a nano EasyLC 1000 (Thermo Fisher Scientific). For this, peptides were loaded onto a reverse-phase C18 (ReproSil-Pur 120 C18-AQ, 1.9 mm, Dr. Maisch GmbH) packed self-made column (75 mm^3^, 150 mm) that was connected to an empty Picotip emitter (New Objective). Solvent compositions in channels A and B were 0.1% FA in H_2_O and 0.1% FA in ACN, respectively. Peptides were injected into the MS at a flow rate of 300 nl/min and were separated using a 120-min gradient of 5 to 35% buffer B. The MS was set to acquire full-scan MS spectra (350 to 1500 mass/charge ratio) at a resolution of 120,000. A top 12 method was used for data-dependent acquisition mode.

Raw files were analyzed using the Proteome Discoverer software v2.4 (Thermo Fisher Scientific). Parent ion and tandem mass spectra were searched against the UniProtKB *Homo sapiens* (UniProt ID UP000005640) and AdV-C5 (UniProt ID UP000004992) databases using the SEQUEST algorithm. For the search, the enzyme specificity was set to trypsin with a maximum of two missed cleavage sites. The precursor mass tolerance was set to 10 parts per million, and the fragment mass tolerance was set to 0.02 Da. Carbamidomethylation of cysteines was set as a fixed modification; N-terminal acetylation, oxidation of methionine, and di-Gly lysines were searched as dynamic modifications. The datasets were filtered on posterior error probability to achieve 1% false discovery rate on protein and peptide level. In addition, spectra containing di-Gly peptides were manually inspected to ensure the presence of a di-Gly remnant on a Lys residue with high confidence. Viral peptides were quantified on the basis of their SILAC label.

### High-pressure freezing and freeze substitution of virus infected samples

Forty thousand HeLa cells were seeded onto 6-mm sapphire disks. Two days after seeding, cells were pretreated with 50 nM LMB, inoculated with either 5 or 25 μg of virus at 37°C for 1 hour, and incubated for an additional 4 hours. Subsequently, cells on sapphire disks were high-pressure frozen, freeze-substituted, and embedded as described previously (*73*). In short, sapphire disks were covered with a 100-μm chamber carrier with 1-hexadecene as a filler and frozen with an EM HPM 100 high-pressure freezing machine (Leica Microsystems, Vienna, Austria). Frozen samples were freeze-substituted in 1% OsO_4_ in anhydrous acetone O/N, block-contrasted with 1% uranyl acetate, and embedded in Epon/Araldite. Ultrathin sections of 70-nm thickness were mounted onto one-slot grids coated with formvar and carbon and post-stained with Reynolds lead citrate.

### Electron microscopy

For the analysis of purified viruses, 5 μl of glycerol-free purified virus was mounted onto carbon-coated grids for 5 min. Grids were washed three times with distilled water and stained for 30 s with 10 μl of a 2% aqueous uranyl acetate solution. Samples were imaged in a CM100 transmission electron microscope at 80 keV (Thermo Fisher Scientific, Eindhoven, The Netherlands).

High-pressure frozen samples were imaged in a Talos 120 transmission electron microscope at an acceleration voltage of 120 kV using a Ceta digital camera and the Maps software package (Thermo Fisher Scientific). Overviews were acquired at a pixel size of 117 nm; detailed imaging of the samples was performed at a pixel size of 0.8 nm with a defocus of −2 μm. Images were quantified manually using Maps Viewer version 3.14.

### Streptolysin O penetration assay

Streptolysin O (SLO) penetration assay was essentially performed as described (*74*). Eighty thousand A549 cells were seeded onto coverslips. On the following day, cells were incubated with ca. 50 bound virus particles of capsid-labeled viruses (ATTO565 or Alexa Fluor 488) on ice for 1 hour. Inocula were washed away with cold binding medium, and cells were incubated at 37°C in a water bath. SLO binding buffer (25 mM Hepes 7.4, 110 mM KOAc, 2.5 mM MgOAC, 0.2 mM CaCl_2_, 1 mM EGTA, and 1 mM DTT) was supplemented with 1 μg of SLO and left at room temperature for 5 min for activation. Cells were incubated in SLO binding buffer with or without SLO for 10 min on ice. Cells were washed twice with SLO binding buffer and incubated for 5 min at 37°C to allow polymerization. Cells were washed once with SLO binding buffer and twice with SLO internalization buffer (25 mM Hepes 7.4, 110 mM KOAc, 2.5 mM MgOAC, and 2 mM EGTA). Cells were incubated with mouse anti-hexon 9C12 or rabbit anti–Alexa Fluor 488 (Molecular Probes) in SLO internalization buffer for 1 hour on ice. Samples were washed three times with SLO internalization buffer and fixed with 3% PFA in 25 mM Hepes-KOH (pH 7.4), 110 mM KOAc, and 2.5 mM MgOAc for 15 min at room temperature. Samples were quenched and stained with secondary antibody goat anti-mouse Alexa Fluor 488 or goat anti-rabbit Alexa Fluor 596 (Thermo Fisher Scientific) diluted in blocking buffer containing DAPI for 1 hour at room temperature. Cells were washed, treated with succinimidyl ester Alexa Fluor 647, and mounted as described above. A Triton X-100 permeabilized sample was included to control the accessibility of the antibodies. Samples were imaged with a Leica SP8 upright confocal microscope. For image analysis, virus particles were segmented according to their capsid label signal and classified as cytosolic on the basis of their antibody staining signal.

### Intracellular trafficking of viral particles

Eighty thousand A549 cells were seeded on coverslips. On the following day, cells were incubated with ca. 80 bound particles of each virus on ice as described above. Inocula were removed, and cells were incubated with infection medium at 37°C. Cells were fixed, quenched, and permeabilized. Samples were stained with mouse anti–hexon 9C12 and goat anti-mouse Alexa Fluor 488 in blocking buffer containing DAPI. Cells were treated with succinimidyl ester Alexa Fluor 647 and mounted onto glass holders. Samples were imaged with a Leica SP8 upright confocal microscope. For image analysis, virus particles were segmented according to their hexon signal and masked with the nuclear signal.

### Thermostability assays

Two micrograms of purified virus particles was incubated in a thermocycler for 5 min at 35°, 40°, 43°, 47°, 50°, 55°, or 60°C. Samples were cooled down on ice and incubated with 5 μM DiYO-1 (AAT Bioquest) for 5 min at room temperature. Fluorescence was recorded with a Tecan Infinite M200 plate reader (excitation, 490 nm; emission, 520 nm). To assess the influence of heat treatment on infection, 10,000 A549 cells were seeded in a black 96-well imaging plate. On the following day, virus suspensions were heat-treated for 5 min at 40°, 40.3°, 40.9°, 42°, 43.4°, 44.8°, 46.2°, 47.6°, 49°, 50°, or 80°C. Samples were placed in infection medium and added to the cells. After 24 hours, cells were fixed, quenched, and permeabilized. Cells were stained with rabbit anti–protein VI and goat anti-rabbit Alexa Fluor 488 in blocking buffer containing DAPI. Cells were imaged in an IXMc high-throughput microscope, and infection was quantified on the basis of the protein VI signal over segmented nuclei.

### Cytokine induction assay

Virus input was normalized to the number of viral particles that bound to MPI-2 cells, which was determined in a binding assay as described above. Four hundred and fifty thousand MPI-2 wild-type, shcGAS, or shEGFP cells (cells expressing shRNA against enhanced GFP) were seeded in a 24-well plate. On the following day, the virus was diluted in cold binding medium to reach 350 bound virus particles and added to the cells for 30 min at 37°C. Inocula were removed; cells were washed two times with cold binding medium, and fresh growth medium was added for a total of 5 or 10 hours of infection. Supernatant was collected, and cells were treated with 200 μl of PBS/1.5 mM EDTA for 3 min and collected along the supernatant. Extracts were centrifuged at 250*g* for 5 min; supernatants were stored at −80°C, and cell pellets were resuspended in 300 μl of TRIzol reagent (Thermo Fisher Scientific). Total RNA was extracted using Direct-zol RNA kit (Zymo Research), and the concentration was measured in a NanoDrop. Two hundred nanograms of purified RNA was reverse-transcribed using Moloney Murine Leukemia Virus Reverse Transcriptase (Promega). RT was primed with oligo dT(15) (Promega) and held for 1 hour at 42°C, followed by a denaturation step for 10 min at 95°C. Samples amplified in the absence of RT were used to control for the presence of genomic DNA. One microliter of the synthesized cDNA was mixed with 0.5 μl of 10 μM premixed primer and SYBR Green JumpStart (Sigma-Aldrich) to reach a final volume of 10 μl and loaded in a MicroAmp Optical 96-well Plate (Applied Biosystems). Forty cycles of amplification at 60°C were conducted in a QuantStudio 3 Real-Time PCR System (Applied Biosystems). Amplification curves were analyzed using QuantStudio Design and Analysis Software v1.5.1. Relative levels of expression compared to a non-infected control were calculated through the 2^ΔΔCt^ method (*75*), using hypoxanthine-guanine phosphoribosyltransferase (HPRT) cDNA as an endogenous loading control. Supernatants collected from these experiments were used for the chemokine and IFNβ production quantifications. CCL2 and CCL5 protein levels were quantified using a sandwich ELISA procedure (Sigma-Aldrich, catalog nos. RAB0055 and RAB0077) following the manufacturer’s instructions. Absorbance at 450 nm was measured in a Tecan Infinite M200 plate reader.

### Type I IFN measurements

Thirty thousand MEF-Mx2-Luc-βKO cells were seeded per well in a 96-well plate. On the following day, MPI-2–derived supernatants were diluted 1:10 and 1:100 in fresh medium and added to the cells for 20 hours. In parallel, recombinant mouse IFNβ (provided by P. Staeheli, University of Freiburg, Germany) was serially diluted and used as a standard to assess IFNβ units per milliliter. Twenty hours after inoculation, supernatants were discarded and cells were washed once with PBS and lysed in 40 μl of 1× CCLR buffer (Promega) for 10 min on a rocking plate at room temperature. Twenty-five microliters from each lysate was transferred to a white Nunclon 96-well plate (Thermo Fisher Scientific). Thirty microliters of Luciferase Assay Reagent (Promega) was added to the wells using a TECAN plate reader with injection unit, followed by shaking for 2 s and integration of the luminescence signal over 10 s.

### RNA FISH with branched DNA signal amplification assay

Forty-five thousand MPI-2 wild-type cells were seeded in a 96-well plate. On the following day, the virus was diluted in cold binding medium to reach 350 bound virus particles and added to the cells for 30 min. Five or 10 hours pi, cells were fixed with 3% PFA in PBS for 30 min at room temperature, washed twice with PBS, and dehydrated by subsequent incubation with 50, 70, and 100% ethanol for 2 min at room temperature. Dehydrated samples were stored at −20°C until staining. Samples were rehydrated by incubation with 70 and 50% ethanol and PBS for 2 min at room temperature. Rehydrated samples were FISH-stained against mouse Ccl2 mRNA using ViewRNA mRNA FISH assay (type 1 probe, Alexa Fluor 546, no. 6006661-01; probes were made against the sequence between mouse Ccl2 gene map positions 2 and 785; Thermo Fisher Scientific) according to the manufacturer’s instructions. Subsequently, cells were incubated in PBS containing DAPI and succinimidyl ester Alexa Fluor 647 (Thermo Fisher Scientific) for 10 min at room temperature. Cells were imaged in an ImageXpress Micro confocal microscope (Molecular Devices) (60-μm pinhole, 15 stacks, and 1.5-μm slice thickness) with a 40× objective. For quantification, cells were segmented according to the DAPI and succinimidyl ester signals, and segmented Ccl2 dots were then related to the cells using CellProfiler (*70*).

### Statistical analysis

All graphs were generated using GraphPad Prism and display means ± SD unless stated otherwise. Statistical tests used are indicated in the figure legends (ns, not significant; *P < 0.05; **P < 0.01; and ***P < 0.001).

## Notes

View/request a protocol for this paper from *Bio-protocol*.

## References

[1] A. S. Lauring, Within-host viral diversity: A window into viral evolution. Annu Rev Virol 7, 63–81 (2020). 32511081 10.1146/annurev-virology-010320-061642 PMC10150642

[2] H. H. Hoffmann, W. M. Schneider, C. M. Rice, Interferons and viruses: An evolutionary arms race of molecular interactions. Trends Immunol. 36, 124–138 (2015). 25704559 10.1016/j.it.2015.01.004 PMC4384471

[3] J. W. Flatt, U. F. Greber, Viral mechanisms for docking and delivering at nuclear pore complexes. Semin. Cell Dev. Biol. 68, 59–71 (2017). 28506891 10.1016/j.semcdb.2017.05.008

[4] L. Lau, E. E. Gray, R. L. Brunette, D. B. Stetson, DNA tumor virus oncogenes antagonize the cGAS-STING DNA-sensing pathway. Science 350, 568–571 (2015). 26405230 10.1126/science.aab3291 PMC12974531

[5] E. Lam, S. Stein, E. Falck-Pedersen, Adenovirus detection by the cGAS/STING/TBK1 DNA densing cascade. J. Virol. 88, 974–981 (2014). 24198409 10.1128/JVI.02702-13 PMC3911663

[6] J. S. Chahal, C. Gallagher, C. J. DeHart, S. J. Flint, The repression domain of the E1B 55-kilodalton protein participates in countering interferon-induced inhibition of adenovirus replication. J. Virol. 87, 4432–4444 (2013). 23388716 10.1128/JVI.03387-12 PMC3624377

[7] G. J. Fonseca, M. J. Cohen, A. C. Nichols, J. W. Barrett, J. S. Mymryk, Viral retasking of hBre1/RNF20 to recruit hPaf1 for transcriptional activation. PLOS Pathog. 9, e1003411 (2013). 23785282 10.1371/journal.ppat.1003411 PMC3681745

[8] O. Takeuchi, S. Akira, Innate immunity to virus infection. Immunol. Rev. 227, 75–86 (2009). 19120477 10.1111/j.1600-065X.2008.00737.x PMC5489343

[9] T. D. Kanneganti, M. Lamkanfi, G. Nunez, Intracellular NOD-like receptors in host defense and disease. Immunity 27, 549–559 (2007). 17967410 10.1016/j.immuni.2007.10.002

[10] A. Ablasser, S. Hur, Regulation of cGAS- and RLR-mediated immunity to nucleic acids. Nat. Immunol. 21, 17–29 (2020). 31819255 10.1038/s41590-019-0556-1

[11] K. A. Karen, P. Hearing, Adenovirus core protein VII protects the viral genome from a DNA damage response at early times after infection. J. Virol. 85, 4135–4142 (2011). 21345950 10.1128/JVI.02540-10 PMC3126275

[12] M. Benevento, S. di Palma, J. Snijder, C. L. Moyer, V. S. Reddy, G. R. Nemerow, A. J. R. Heck, Adenovirus composition, proteolysis, and disassembly studied by in-depth qualitative and quantitative proteomics. J. Biol. Chem. 289, 11421–11430 (2014). 24591515 10.1074/jbc.M113.537498 PMC4036278

[13] L. C. Trotman, N. Mosberger, M. Fornerod, R. P. Stidwill, U. F. Greber, Import of adenovirus DNA involves the nuclear pore complex receptor CAN/Nup214 and histone H1. Nat. Cell Biol. 3, 1092–1100 (2001). 11781571 10.1038/ncb1201-1092

[14] H. Wodrich, A. Cassany, M. A. D’Angelo, T. Guan, G. Nemerow, L. Gerace, Adenovirus core protein pVII is translocated into the nucleus by multiple import receptor pathways. J. Virol. 80, 9608–9618 (2006). 16973564 10.1128/JVI.00850-06 PMC1617226

[15] U. F. Greber, M. Suomalainen, R. P. Stidwill, K. Boucke, M. W. Ebersold, A. Helenius, The role of the nuclear pore complex in adenovirus DNA entry. EMBO J. 16, 5998–6007 (1997). 9312057 10.1093/emboj/16.19.5998 PMC1170230

[16] J. Chen, N. Morral, D. A. Engel, Transcription releases protein VII from adenovirus chromatin. Virology 369, 411–422 (2007). 17888479 10.1016/j.virol.2007.08.012

[17] I. H. Wang, M. Suomalainen, V. Andriasyan, S. Kilcher, J. Mercer, A. Neef, N. W. Luedtke, U. F. Greber, Tracking viral genomes in host cells at single-molecule resolution. Cell Host Microbe 14, 468–480 (2013). 24139403 10.1016/j.chom.2013.09.004

[18] D. Puntener, M. F. Engelke, Z. Ruzsics, S. Strunze, C. Wilhelm, U. F. Greber, Stepwise loss of fluorescent core protein V from human adenovirus during entry into cells. J. Virol. 85, 481–496 (2011). 21047958 10.1128/JVI.01571-10 PMC3014209

[19] S. Strunze, M. F. Engelke, I. H. Wang, D. Puntener, K. Boucke, S. Schleich, M. Way, P. Schoenenberger, C. J. Burckhardt, U. F. Greber, Kinesin-1-mediated capsid disassembly and disruption of the nuclear pore complex promote virus infection. Cell Host Microbe 10, 210–223 (2011). 21925109 10.1016/j.chom.2011.08.010

[20] D. Komander, M. Rape, The ubiquitin code. Annu. Rev. Biochem. 81, 203–229 (2012). 22524316 10.1146/annurev-biochem-060310-170328

[21] A. J. George, Y. C. Hoffiz, A. J. Charles, Y. Zhu, A. M. Mabb, A comprehensive atlas of E3 ubiquitin ligase mutations in neurological disorders. Front. Genet. 9, 29 (2018). 29491882 10.3389/fgene.2018.00029 PMC5817383

[22] M. K. Isaacson, H. L. Ploegh, Ubiquitination, ubiquitin-like modifiers, and deubiquitination in viral infection. Cell Host Microbe 5, 559–570 (2009). 19527883 10.1016/j.chom.2009.05.012 PMC7103382

[23] F. Randow, P. J. Lehner, Viral avoidance and exploitation of the ubiquitin system. Nat. Cell Biol. 11, 527–534 (2009). 19404332 10.1038/ncb0509-527

[24] I. Banerjee, Y. Miyake, S. P. Nobs, C. Schneider, P. Horvath, M. Kopf, P. Matthias, A. Helenius, Y. Yamauchi, Influenza A virus uses the aggresome processing machinery for host cell entry. Science 346, 473–477 (2014). 25342804 10.1126/science.1257037

[25] H. Wodrich, D. Henaff, B. Jammart, C. Segura-Morales, S. Seelmeir, O. Coux, Z. Ruzsics, C. M. Wiethoff, E. J. Kremer, A capsid-encoded PPxY-motif facilitates adenovirus entry. PLOS Pathog. 6, e1000808 (2010). 20333243 10.1371/journal.ppat.1000808 PMC2841620

[26] J. O. Akello, R. Kamgang, M. T. Barbani, F. Suter-Riniker, S. L. Leib, A. Ramette, Epidemiology of human adenoviruses: A 20-year retrospective observational study in hospitalized patients in Bern, Switzerland. Clin. Epidemiol. 12, 353–366 (2020). 32308491 10.2147/CLEP.S246352 PMC7147615

[27] A. J. Colom, A. M. Teper, Post-infectious bronchiolitis obliterans. Pediatr. Pulmonol. 54, 212–219 (2019). 30548423 10.1002/ppul.24221

[28] T. Lion, Adenovirus persistence, reactivation, and clinical management. FEBS Lett. 593, 3571–3582 (2019). 31411731 10.1002/1873-3468.13576

[29] V. Prasad, M. Suomalainen, Y. Jasiqi, S. Hemmi, P. Hearing, L. Hosie, H. G. Burgert, U. F. Greber, The UPR sensor IRE1α and the adenovirus E3-19K glycoprotein sustain persistent and lytic infections. Nat. Commun. 11, 1997 (2020). 32332742 10.1038/s41467-020-15844-2 PMC7181865

[30] F. C. Zhu, Y.-H. Li, X.-H. Guan, L.-H. Hou, W.-J. Wang, J.-X. Li, S.-P. Wu, B.-S. Wang, Z. Wang, L. Wang, S.-Y. Jia, H.-D. Jiang, L. Wang, T. Jiang, Y. Hu, J.-B. Gou, S.-B. Xu, J.-J. Xu, X.-W. Wang, W. Wang, W. Chen, Safety, tolerability, and immunogenicity of a recombinant adenovirus type-5 vectored COVID-19 vaccine: A dose-escalation, open-label, non-randomised, first-in-human trial. Lancet 13, 1845–1854 (2020). 10.1016/S0140-6736(20)31208-3 PMC7255193 32450106

[31] U. F. Greber, Adenoviruses—Infection, pathogenesis and therapy. FEBS Lett. 594, 1818–1827 (2020). 32538496 10.1002/1873-3468.13849

[32] M. N. Ramasamy, A. M. Minassian, K. J. Ewer, A. L. Flaxman, P. M. Folegatti, D. R. Owens, M. Voysey, P. K. Aley, B. Angus, G. Babbage, S. Belij-Rammerstorfer, L. Berry, S. Bibi, M. Bittaye, K. Cathie, H. Chappell, S. Charlton, P. Cicconi, E. A. Clutterbuck, R. Colin-Jones, C. Dold, K. R. W. Emary, S. Fedosyuk, M. Fuskova, D. Gbesemete, C. Green, B. Hallis, M. M. Hou, D. Jenkin, C. C. D. Joe, E. J. Kelly, S. Kerridge, A. M. Lawrie, A. Lelliott, M. N. Lwin, R. Makinson, N. G. Marchevsky, Y. Mujadidi, A. P. S. Munro, M. Pacurar, E. Plested, J. Rand, T. Rawlinson, S. Rhead, H. Robinson, A. J. Ritchie, A. L. Ross-Russell, S. Saich, N. Singh, C. C. Smith, M. D. Snape, R. Song, R. Tarrant, Y. Themistocleous, K. M. Thomas, T. L. Villafana, S. C. Warren, M. E. E. Watson, A. D. Douglas, A. V. S. Hill, T. Lambe, S. C. Gilbert, S. N. Faust, A. J. Pollard; Oxford COVID Vaccine Trial Group, Safety and immunogenicity of ChAdOx1 nCoV-19 vaccine administered in a prime-boost regimen in young and old adults (COV002): A single-blind, randomised, controlled, phase 2/3 trial. Lancet 396, 1979–1993 (2021). 33220855 10.1016/S0140-6736(20)32466-1 PMC7674972

[33] B. Guo, B. J. McMillan, S. C. Blacklow, Structure and function of the Mind bomb E3 ligase in the context of Notch signal transduction. Curr. Opin. Struct. Biol. 41, 38–45 (2016). 27285058 10.1016/j.sbi.2016.05.012 PMC5143217

[34] B. H. Villumsen, J. R. Danielsen, L. Povlsen, K. B. Sylvestersen, A. Merdes, P. Beli, Y. G. Yang, C. Choudhary, M. L. Nielsen, N. Mailand, S. Bekker-Jensen, A new cellular stress response that triggers centriolar satellite reorganization and ciliogenesis. EMBO J. 32, 3029–3040 (2013). 24121310 10.1038/emboj.2013.223 PMC3844950

[35] M. Bauer, J. W. Flatt, D. Seiler, B. Cardel, M. Emmenlauer, K. Boucke, M. Suomalainen, S. Hemmi, U. F. Greber, The E3 ubiquitin ligase Mind bomb 1 controls adenovirus genome release at the nuclear pore complex. Cell Rep. 29, 3785–3795.e8 (2019). 31851912 10.1016/j.celrep.2019.11.064

[36] S. L. Sarbanes, V. A. Blomen, E. Lam, S. Heissel, J. M. Luna, T. R. Brummelkamp, E. Falck-Pedersen, H. H. Hoffmann, C. M. Rice, E3 ubiquitin ligase Mindbomb 1 facilitates nuclear delivery of adenovirus genomes. Proc. Natl. Acad. Sci. U.S.A. 118, e2015794118 (2021). 33443154 10.1073/pnas.2015794118 PMC7817214

[37] H. Ugai, G. C. Dobbins, M. Wang, L. P. le, D. A. Matthews, D. T. Curiel, Adenoviral protein V promotes a process of viral assembly through nucleophosmin 1. Virology 432, 283–295 (2012). 22717133 10.1016/j.virol.2012.05.028 PMC3423539

[38] V. S. Reddy, G. R. Nemerow, Structures and organization of adenovirus cement proteins provide insights into the role of capsid maturation in virus entry and infection. Proc. Natl. Acad. Sci. U.S.A. 111, 11715–11720 (2014). 25071205 10.1073/pnas.1408462111 PMC4136627

[39] M. Suomalainen, V. Prasad, A. Kannan, U. F. Greber, Cell-to-cell and genome-to-genome variability of adenovirus transcription tuned by the cell cycle. J. Cell Sci. 134, jcs252544 (2020). 32917739 10.1242/jcs.252544

[40] I. H. Wang, C. J. Burckhardt, A. Yakimovich, M. K. Morf, U. F. Greber, The nuclear export factor CRM1 controls juxta-nuclear microtubule-dependent virus transport. J. Cell Sci. 130, 2185–2195 (2017). 28515232 10.1242/jcs.203794

[41] N. Stichling, M. Suomalainen, J. W. Flatt, M. Schmid, M. Pacesa, S. Hemmi, W. Jungraithmayr, M. D. Maler, M. A. Freudenberg, A. Plückthun, T. May, M. Köster, G. Fejer, U. F. Greber, Lung macrophage scavenger receptor SR-A6 (MARCO) is an adenovirus type-specific virus entry receptor. PLOS Pathog. 14, e1006914 (2018). 29522575 10.1371/journal.ppat.1006914 PMC5862501

[42] M. Nociari, O. Ocheretina, J. W. Schoggins, E. Falck-Pedersen, Sensing infection by adenovirus: Toll-like receptor-independent viral DNA recognition signals activation of the interferon regulatory factor 3 master regulator. J. Virol. 81, 4145–4157 (2007). 17251283 10.1128/JVI.02685-06 PMC1866159

[43] G. Xu, J. S. Paige, S. R. Jaffrey, Global analysis of lysine ubiquitination by ubiquitin remnant immunoaffinity profiling. Nat. Biotechnol. 28, 868–873 (2010). 20639865 10.1038/nbt.1654 PMC2946519

[44] Z. Cheng, T. Dai, X. He, Z. Zhang, F. Xie, S. Wang, L. Zhang, F. Zhou, The interactions between cGAS-STING pathway and pathogens. Signal Transduct. Target. Ther. 5, 91 (2020). 32532954 10.1038/s41392-020-0198-7 PMC7293265

[45] X. Lahaye, T. Satoh, M. Gentili, S. Cerboni, C. Conrad, I. Hurbain, A. el Marjou, C. Lacabaratz, J. D. Lelièvre, N. Manel, The capsids of HIV-1 and HIV-2 determine immune detection of the viral cDNA by the innate sensor cGAS in dendritic cells. Immunity 39, 1132–1142 (2013). 24269171 10.1016/j.immuni.2013.11.002

[46] S. Gallucci, M. E. Maffei, DNA sensing across the tree of life. Trends Immunol. 38, 719–732 (2017). 28886908 10.1016/j.it.2017.07.012

[47] S. Kulanayake, S. K. Tikoo, Adenovirus core proteins: Structure and function. Viruses 13, (2021). 10.3390/v13030388 PMC7998265 33671079

[48] C. C. de Oliveira Mann, M. H. Orzalli, D. S. King, J. C. Kagan, A. S. Y. Lee, P. J. Kranzusch, Modular architecture of the STING C-terminal tail allows interferon and NF-κB signaling adaptation. Cell Rep. 27, 1165–1175.e5 (2019). 31018131 10.1016/j.celrep.2019.03.098 PMC7733315

[49] M. Du, Z. J. Chen, DNA-induced liquid phase condensation of cGAS activates innate immune signaling. Science 361, 704–709 (2018). 29976794 10.1126/science.aat1022 PMC9417938

[50] P. K. Chatterjee, M. E. Vayda, S. J. Flint, Interactions among the three adenovirus core proteins. J. Virol. 55, 379–386 (1985). 4020954 10.1128/jvi.55.2.379-386.1985 PMC254944

[51] J. Perez-Vargas, R. C. Vaughan, C. Houser, K. M. Hastie, C. C. Kao, G. R. Nemerow, Isolation and characterization of the DNA and protein binding activities of adenovirus core protein V. J. Virol. 88, 9287–9296 (2014). 24899200 10.1128/JVI.00935-14 PMC4136271

[52] L. I. Labzin, M. Bottermann, P. Rodriguez-Silvestre, S. Foss, J. T. Andersen, M. Vaysburd, D. Clift, L. C. James, Antibody and DNA sensing pathways converge to activate the inflammasome during primary human macrophage infection. EMBO J. 38, e101365 (2019). 31468569 10.15252/embj.2018101365 PMC6826209

[53] I. H. Wang, C. J. Burckhardt, A. Yakimovich, U. F. Greber, Imaging, tracking and computational analyses of virus entry and egress with the cytoskeleton. Viruses 10, 166 (2018). 29614729 10.3390/v10040166 PMC5923460

[54] J. Zhou, J. Scherer, J. Yi, R. B. Vallee, Role of kinesins in directed adenovirus transport and cytoplasmic exploration. PLOS Pathog. 14, e1007055 (2018). 29782552 10.1371/journal.ppat.1007055 PMC5983873

[55] O. Kobiler, N. Drayman, V. Butin-Israeli, A. Oppenheim, Virus strategies for passing the nuclear envelope barrier. Nucleus 3, 526–539 (2012). 22929056 10.4161/nucl.21979 PMC3515536

[56] S. R. Wente, M. P. Rout, The nuclear pore complex and nuclear transport. Cold Spring Harb. Perspect. Biol. 2, a000562 (2010). 20630994 10.1101/cshperspect.a000562 PMC2944363

[57] W. Kim, E. J. Bennett, E. L. Huttlin, A. Guo, J. Li, A. Possemato, M. E. Sowa, R. Rad, J. Rush, M. J. Comb, J. W. Harper, S. P. Gygi, Systematic and quantitative assessment of the ubiquitin-modified proteome. Mol. Cell 44, 325–340 (2011). 21906983 10.1016/j.molcel.2011.08.025 PMC3200427

[58] S. Bergstrom Lind, K. A. Artemenko, L. Elfineh, Y. Zhao, J. Bergquist, U. Pettersson, Post translational modifications in adenovirus type 2. Virology 447, 104–111 (2013). 24210104 10.1016/j.virol.2013.08.033

[59] J. K. Gustin, A. V. Moses, K. Fruh, J. L. Douglas, Viral takeover of the host ubiquitin system. Front. Microbiol. 2, 161 (2011). 21847386 10.3389/fmicb.2011.00161 PMC3147166

[60] B. Harrach, Z. L. Tarjan, M. Benko, Adenoviruses across the animal kingdom: A walk in the zoo. FEBS Lett. 593, 3660–3673 (2019). 31747467 10.1002/1873-3468.13687

[61] C. M. Robinson, G. Singh, J. Y. Lee, S. Dehghan, J. Rajaiya, E. B. Liu, M. A. Yousuf, R. A. Betensky, M. S. Jones, D. W. Dyer, D. Seto, J. Chodosh, Molecular evolution of human adenoviruses. Sci. Rep. 3, 1812 (2013). 23657240 10.1038/srep01812 PMC3648800

[62] K. Peng, W. Muranyi, B. Glass, V. Laketa, S. R. Yant, L. Tsai, T. Cihlar, B. Müller, H. G. Kräusslich, Quantitative microscopy of functional HIV post-entry complexes reveals association of replication with the viral capsid. eLife 3, (2014). 10.7554/eLife.04114 PMC4293571 25517934

[63] D. Goubau, S. Deddouche, E. S. C. Reis, Cytosolic sensing of viruses. Immunity 38, 855–869 (2013). 23706667 10.1016/j.immuni.2013.05.007 PMC7111113

[64] G. Fejer, M. D. Wegner, I. Gyory, I. Cohen, P. Engelhard, E. Voronov, T. Manke, Z. Ruzsics, L. Dolken, O. Prazeres da Costa, N. Branzk, M. Huber, A. Prasse, R. Schneider, R. N. Apte, C. Galanos, M. A. Freudenberg, Nontransformed, GM-CSF-dependent macrophage lines are a unique model to study tissue macrophage functions. Proc. Natl. Acad. Sci. U.S.A. 110, E2191–E2198 (2013). 23708119 10.1073/pnas.1302877110 PMC3683787

[65] U. F. Greber, M. Willetts, P. Webster, A. Helenius, Stepwise dismantling of adenovirus 2 during entry into cells. Cell 75, 477–486 (1993). 8221887 10.1016/0092-8674(93)90382-z

[66] P. Hearing, T. Shenk, The adenovirus type 5 E1A transcriptional control region contains a duplicated enhancer element. Cell 33, 695–703 (1983). 6871991 10.1016/0092-8674(83)90012-0

[67] W. W. Colby, T. Shenk, Adenovirus type 5 virions can be assembled in vivo in the absence of detectable polypeptide IX. J. Virol. 39, 977–980 (1981). 7288921 10.1128/jvi.39.3.977-980.1981 PMC171335

[68] C. J. Burckhardt, M. Suomalainen, P. Schoenenberger, K. Boucke, S. Hemmi, U. F. Greber, Drifting motions of the adenovirus receptor CAR and immobile integrins initiate virus uncoating and membrane lytic protein exposure. Cell Host Microbe 10, 105–117 (2011). 21843868 10.1016/j.chom.2011.07.006

[69] S. Warming, N. Costantino, D. L. Court, N. A. Jenkins, N. Copeland, Simple and highly efficient BAC recombineering using galK selection. Nucleic Acids Res. 33, e36 (2005). 15731329 10.1093/nar/gni035 PMC549575

[70] A. E. Carpenter, T. R. Jones, M. R. Lamprecht, C. Clarke, I. Kang, O. Friman, D. A. Guertin, J. Chang, R. A. Lindquist, J. Moffat, P. Golland, D. M. Sabatini, CellProfiler: Image analysis software for identifying and quantifying cell phenotypes. Genome Biol. 7, R100 (2006). 17076895 10.1186/gb-2006-7-10-r100 PMC1794559

[71] R. Varghese, Y. Mikyas, P. L. Stewart, R. Ralston, Postentry neutralization of adenovirus type 5 by an antihexon antibody. J. Virol. 78, 12320–12332 (2004). 15507619 10.1128/JVI.78.22.12320-12332.2004 PMC525062

[72] N. D. Udeshi, P. Mertins, T. Svinkina, S. A. Carr, Large-scale identification of ubiquitination sites by mass spectrometry. Nat. Protoc. 8, 1950–1960 (2013). 24051958 10.1038/nprot.2013.120 PMC4725055

[73] A. Kaech, U. Ziegler, High-pressure freezing: Current state and future prospects. Methods Mol. Biol. 1117, 151–171 (2014). 24357363 10.1007/978-1-62703-776-1_8

[74] M. Suomalainen, S. Luisoni, K. Boucke, S. Bianchi, D. A. Engel, U. F. Greber, A direct and versatile assay measuring membrane penetration of adenovirus in single cells. J. Virol. 87, 12367–12379 (2013). 24027314 10.1128/JVI.01833-13 PMC3807902

[75] T. D. Schmittgen, K. J. Livak, Analyzing real-time PCR data by the comparative C(T) method. Nat. Protoc. 3, 1101–1108 (2008). 18546601 10.1038/nprot.2008.73

