# Supplementary material for: A viral ubiquitination switch attenuates innate immunity and triggers nuclear import of virion DNA and infection
Source: Sci Adv. 2021 Dec 17;7(51):eabl7150. doi: 10.1126/sciadv.abl7150 (PMC8682987; doi:10.1126/sciadv.abl7150)
Supplement: Supplementary file 1 — Figs. S1 to S5 Tables S1 and S2 Legend for movie S1 Legends for data S1 and S2 [file sciadv.abl7150_sm.pdf]

Science Advances
AAAS

Supplementary Materials for

A viral ubiquitination switch attenuates innate immunity and triggers nuclear import of virion DNA and infection

Michael Bauer, Alfonso Gomez-Gonzalez, Maarit Suomalainen, Nicolas Schilling, Silvio Hemmi, Urs F. Greber*

*Corresponding author. Email: urs.greber@mls.uzh.ch

Published 17 December 2021, *Sci. Adv.* **7**, eabl7150 (2021)
DOI: 10.1126/sciadv.abl7150

The PDF file includes:

Figs. S1 to S5
Tables S1 and S2
Legend for movie S1
Legends for data S1 and S2

Other Supplementary Material for this manuscript includes the following:

Movie S1
Data S1 and S2

Supplementary Figures

Figure S1

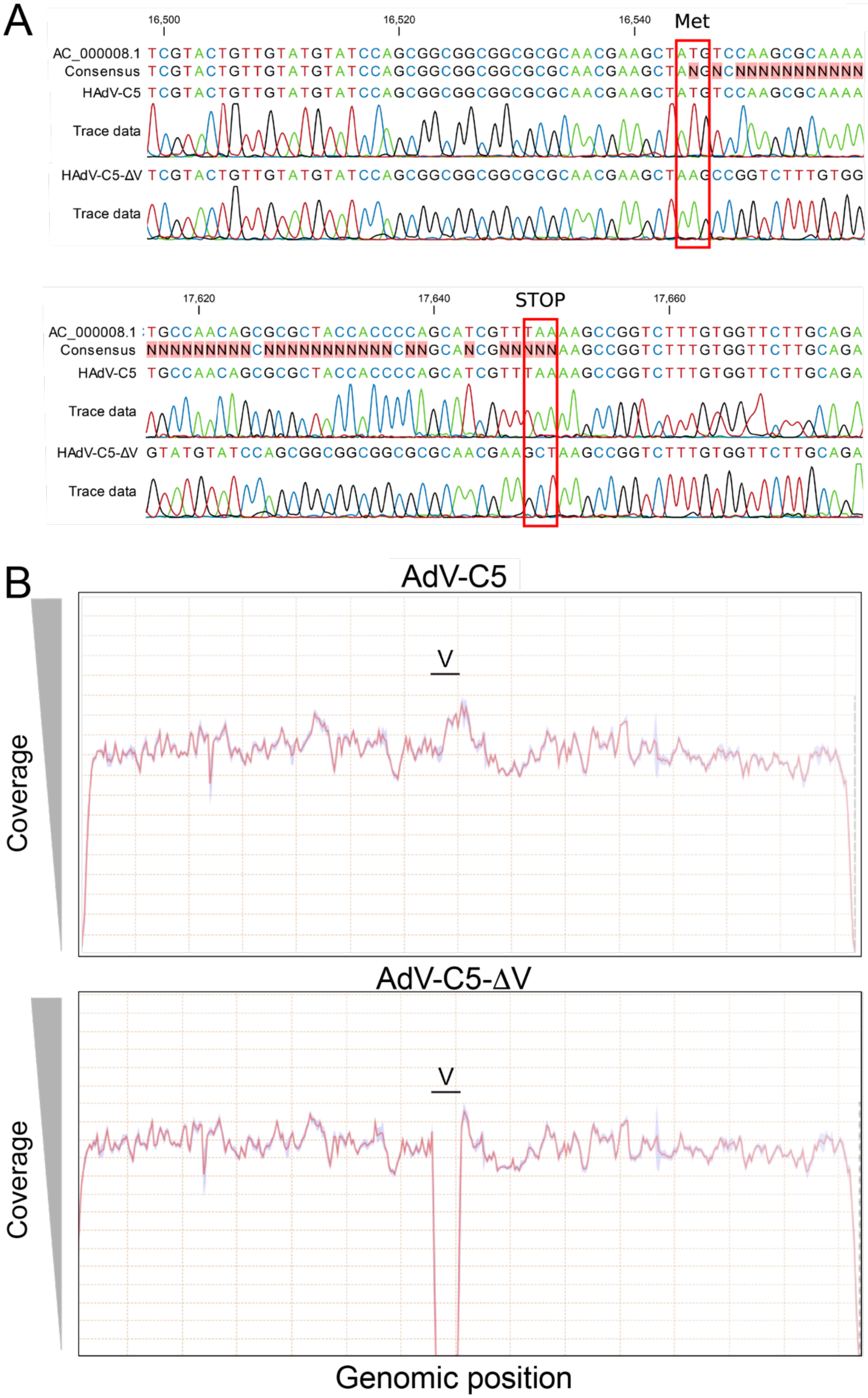

Fig. S1: Characterization of AdV-C5-ΔV (refers to Fig. 1). (**A**) Sequencing analysis of AdV-C5-ΔV. The protein V genomic region was PCR amplified from purified AdV-C5 and AdV-C5-ΔV viral genomes. Band purified PCR amplicons were sequenced using two internal primers. Sequences were aligned to wild-type AC_000008.1 reference. Non-matching nucleotides are highlighted with red in the consensus sequence. (**B**) Mapping of short read Illumina NGS runs to the AC_000008.1 reference using a Bowtie2 algorithm. Nucleotide coverage is displayed in function of AC_000008.1 genomic position for both AdV-C5 and AdV-C5-ΔV. (**C**) AdV particles lacking protein V are less infectious than the wild-type particles. HeLa cells were inoculated with AdV-C5 or AdV-C5-ΔV for 1h on ice, yielding a range of 0.05 to 50 bound viral particles per cell. The inoculum was replaced by fresh medium and cells incubated at 37°C for 20h or 40h, fixed and stained with anti-VI antibody and DAPI (nucleus). Infection was scored by percentage of protein VI-positive nuclei. Data show the means ± SD.

Figure S2

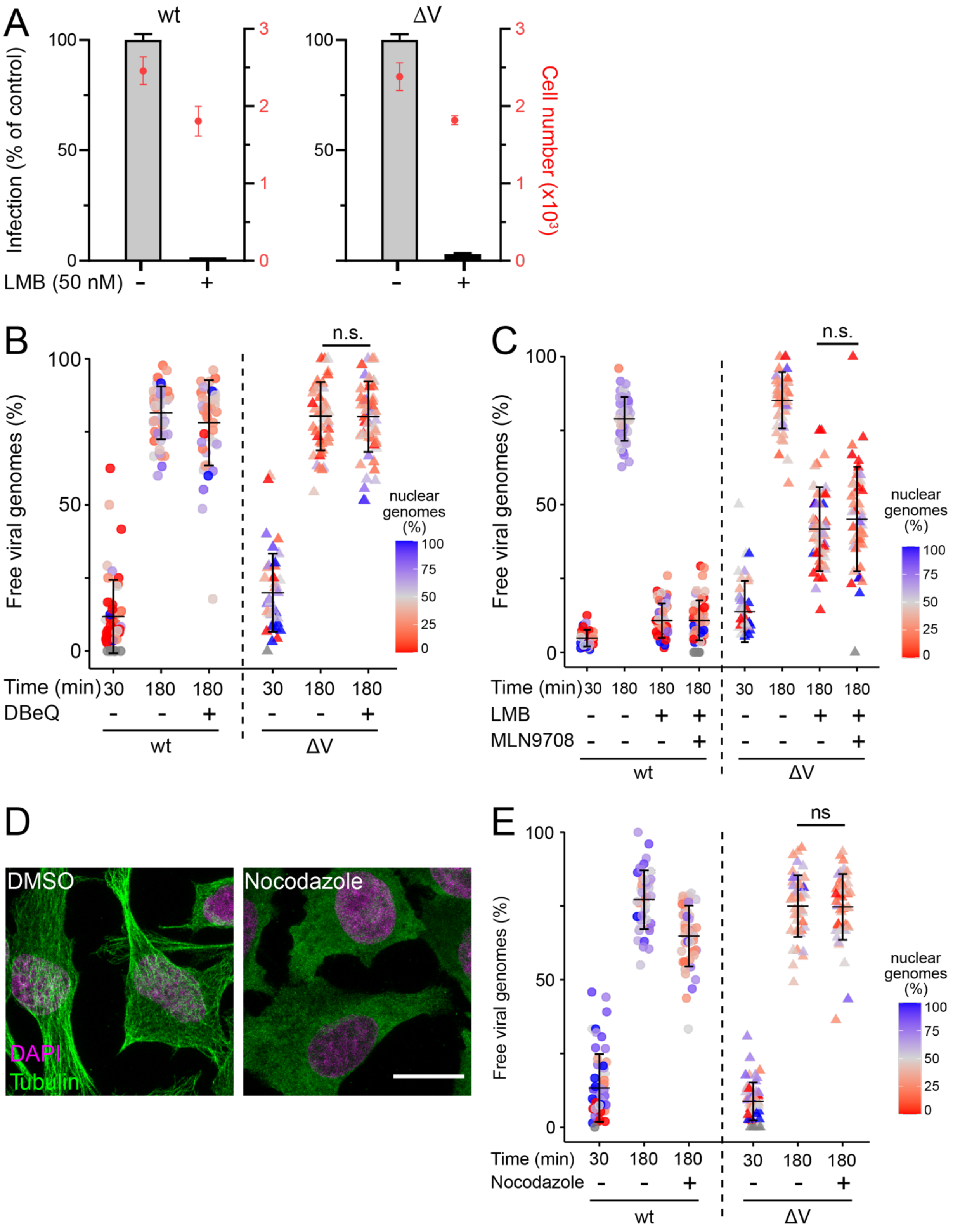
A
wt
ΔV
Infection (% of control)
Cell number (x10³)
100
50
0
3
2
1
0
LMB (50 nM)
-
+
B
n.s.
Free viral genomes (%)
nuclear genomes (%)
100
75
50
25
0
Time (min) 30 180 180 30 180 180
DBeQ - - + - - +
wt
ΔV
C
n.s.
Free viral genomes (%)
nuclear genomes (%)
Time (min) 30 180 180 180 30 180 180 180
LMB - - + + - - + +
MLN9708 - - - + - - - +
wt
ΔV
D
DMSO
Nocodazole
DAPI
Tubulin
E
ns
Free viral genomes (%)
nuclear genomes (%)
Time (min) 30 180 180 30 180 180
Nocodazole - - + - - +
wt
ΔV

Fig. S2: Premature genome release from AdV-C5-ΔV is independent of p97, the proteasome, and microtubules (refers to Fig. 3). (**A**) LMB blocks AdV-C5-ΔV infection. HeLa cells were infected with AdV-C5 or AdV-C5-ΔV at a MOI of 0.5 for 24h in the presence of vehicle or 50 nM LMB. After fixation, cells were stained with anti-pVI antibody and DAPI. Infection was scored by percentage of pVI-positive nuclei. Graphs show the mean ± SD. (**B**) HeLa cells were incubated with genome-labeled AdV-C5 or AdV-C5-ΔV for 30 or 180 min in the absence or presence of 5 μM DBeQ. Cells were fixed and stained with anti-Hexon and vDNA using click chemistry. Data are shown as mean ± SD. Ratio of the number of capsid-free genomes over the nuclear mask has been color-coded. Statistical significance was assessed by using a non-parametric ANOVA (Kruskal-Wallis test) with Dunn's correction for multiple comparisons. ns, not significant. (**C**) HeLa cells were incubated with genome-labeled AdV-C5 or AdV-C5-ΔV for 30 or 180 min in the absence or presence of 50 nM LMB and 10 μM MLN9708. Cells were fixed and stained with anti-Hexon and vDNA using click chemistry. Data are shown as mean ± SD. Ratio of the number of capsid-free genomes over the nuclear mask has been color-coded. Statistical significance was assessed as in (B). (**D, E**) HeLa cells were incubated with genome-labeled AdV-C5 or AdV-C5-ΔV in the absence or presence of 10 nM Nocodazole. Data are shown as mean ± SD. Ratio of the number of capsid-free genomes over the nuclear mask has been color-coded. Statistical significance was assessed as in (B). Tubulin staining shows depolymerization of microtubules. Images are maximum projections. Scale bar, 10 μm.

Figure S3

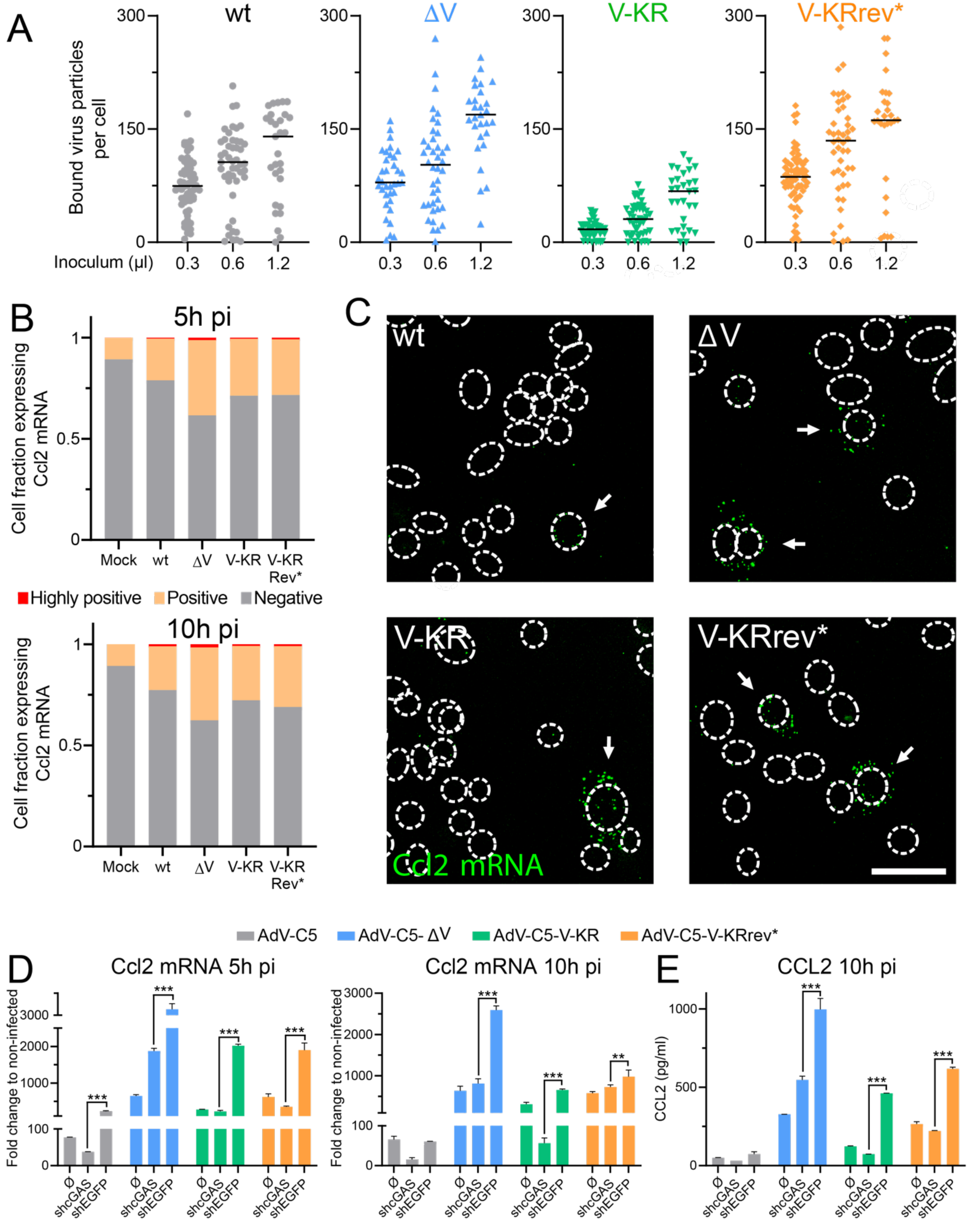

Fig. S3: Protein V mutants induce stronger cytokine response than wild-type virus (refers to Fig. 4). (**A**) Imaging-based quantification of AdV-C5 inoculated

to MPI-2 cells. MPI-2 cells were inoculated with different amounts of AdV-C5, AdV-C5-ΔV, AdV-C5-V-KR or AdV-C5-V-KRrev* at 37°C for 30 min, inoculum washed away, samples fixed with 3% PFA, and stained with anti-hexon, DAPI, and AlexaFluor 647-conjugated succinimidyl ester, the latter to delineate cell outline. Virus particles were segmented and assessed on a per cell basis. Data show the median (black bars). **, p < 0.01. (**B, C**) MPI-2 cells were infected with AdV-C5, AdV-C5-ΔV, AdV-C5-V-KR or AdV-C5-V-KRrev* for 5 or 10h. Cells were fixed and stained against Ccl2 mRNA using RNA FISH with branched DNA signal amplification. Nuclei were stained with DAPI and cell outlines with AlexaFluor 647-conjugated succinimidyl ester. Ccl2 mRNA dots were segmented and quantified per cell. Highly positive cells contain more than 10 transcripts per cell. Images are maximum projections. Nuclear outlines are based on DAPI. Arrows indicate high Ccl2 expressing cells. Scale bar, 20 µm. (**D**) MPI-2 wt, shcGAS, or shEGFP cells were inoculated with AdV-C5, AdV-C5-ΔV, AdV-C5-V-KR or AdV-C5-V-KRrev* for 5 or 10h, lysed and processed for RT-qPCR. Fold change in Ccl2 mRNA expression was addressed through the 2^-(ΔΔCt) method using HPRT as an endogenous control. Statistical significance was assessed by one-way ANOVA with Holm-Sidak correction for multiple comparisons. ***, p<0.001. (**E**) Supernatant from MPI-2 wt, shcGAS or shEGFP cells mock or infected with AdV-C5, AdV-C5-ΔV, AdV-C5-V-KR or AdV-C5-V-KRrev* were quantified with Sigma's Mouse CCL2 ELISA Kit according to the manufacturer's instructions. Statistical significance was assessed as in (D).

Figure S4

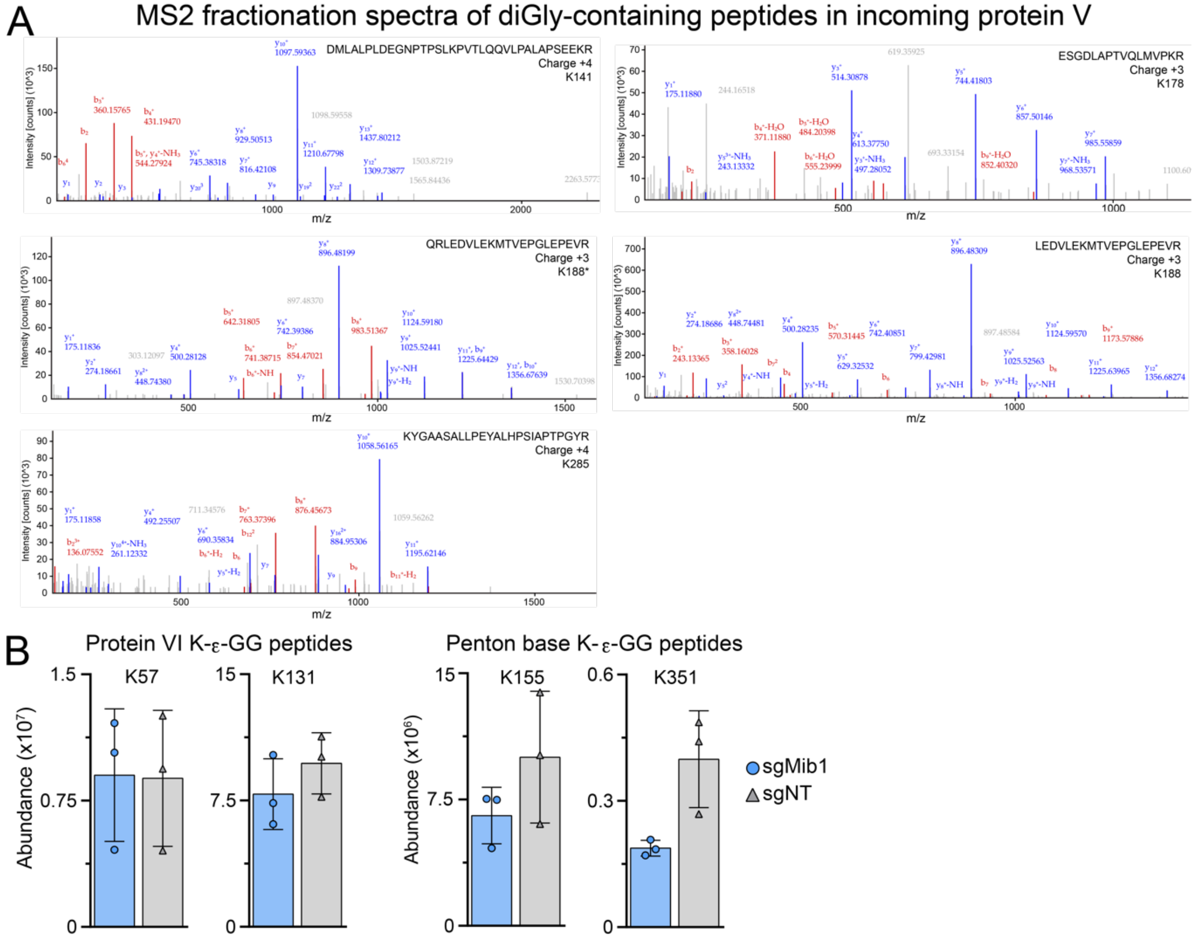

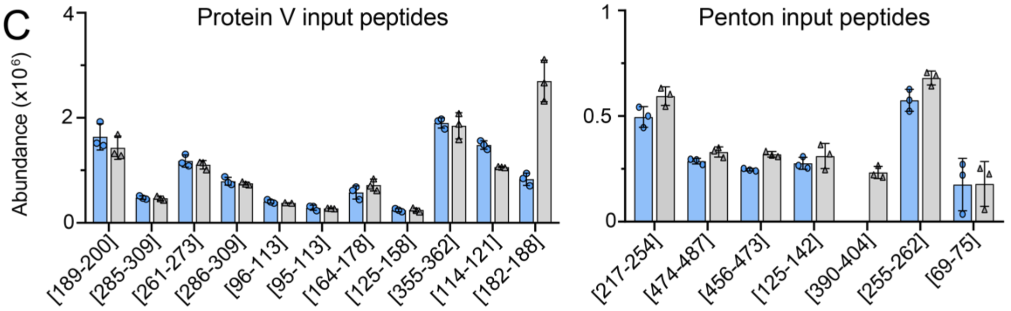

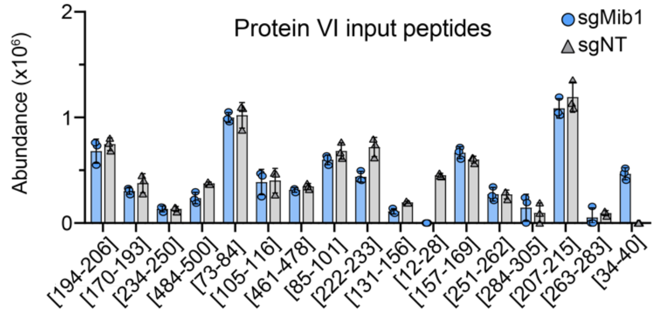

D LC-MS/MS of purified viral particles

Accession number	Protein	Coverage (%)	Identified peptides	Peptides cont. K-ε-GG
**P24938**	Protein V	63	35	-
**P68951**	Protein VII	62	18	-
**P12537**	Protein IIIa	70	39	-
**P24936**	Protein VIII	49	10	-
**P04133**	Hexon	49	43	-
**P24937**	Protein VI	42	12	-
**P12538**	Penton	44	24	-
**P03281**	Protein IX	84	6	-
**P03253**	Protease	51	13	
**P04499**	Terminal protein	16	11	-
**P03271**	Packaging protein IVa2	19	8	-
**P11818**	Fiber	16	7	-
**P14269**	Protein X	19	3	-

Fig. S4: Identification of protein V ubiquitination sites by LC-MS/MS and peptide abundance of K-ε-GG-containing viral proteins (refers to Fig. 5). (**A**) HeLa-sgNT cells and HeLa-sgMib1 cells were incubated with light or heavy AdV-C5, respectively, for 2h. After lysis and di-glycine immunoprecipitation, ubiquitinated peptides were analyzed by mass spectrometry (MS). AdV protein V ubiquitination sites were identified following trypsin digestion and LC MS/MS analysis. Four ubiquitination sites were detected, including K141, K178, K188, and K285. The sites were identified with Proteome Discoverer where the FDR was restricted to 1% on peptide and site level. The Lys residue carrying the diGly remnant is indicated in red. The peptide containing K188 annotated with an asterisk is the result of a missed cleavage. All matching y and b ions are annotated with their measured m/z (mass to charge). (**B**) Abundance of two representative peptides of protein VI and the two peptides of penton base containing K-ε-GG residues. Lysine residue containing GG within each peptide is indicated on top of each graph. (**C**) Aliquots of the samples collected in (A) were analyzed without enrichment of di-glycine peptides to ensure comparable amounts of inocula. Abundance of identified AdV-C5 protein VI, protein V, and penton base peptides was highly comparable. (**D**) Cesium chloride gradient purified heavy AdV-C5-wt particles were lysed, alkylated, and trypsin digested. Peptides were desalted and analyzed by LC-MS/MS. Peptide identification was performed with Mascot 2.6. Carbamidomethylation of cysteines was set as a fixed modification; variable modifications were diGly(K), N-terminal acetylation, and oxidation of methionines. None of the identified peptides of the virion contained a diGly remnant on a lysine residue.

Figure S5

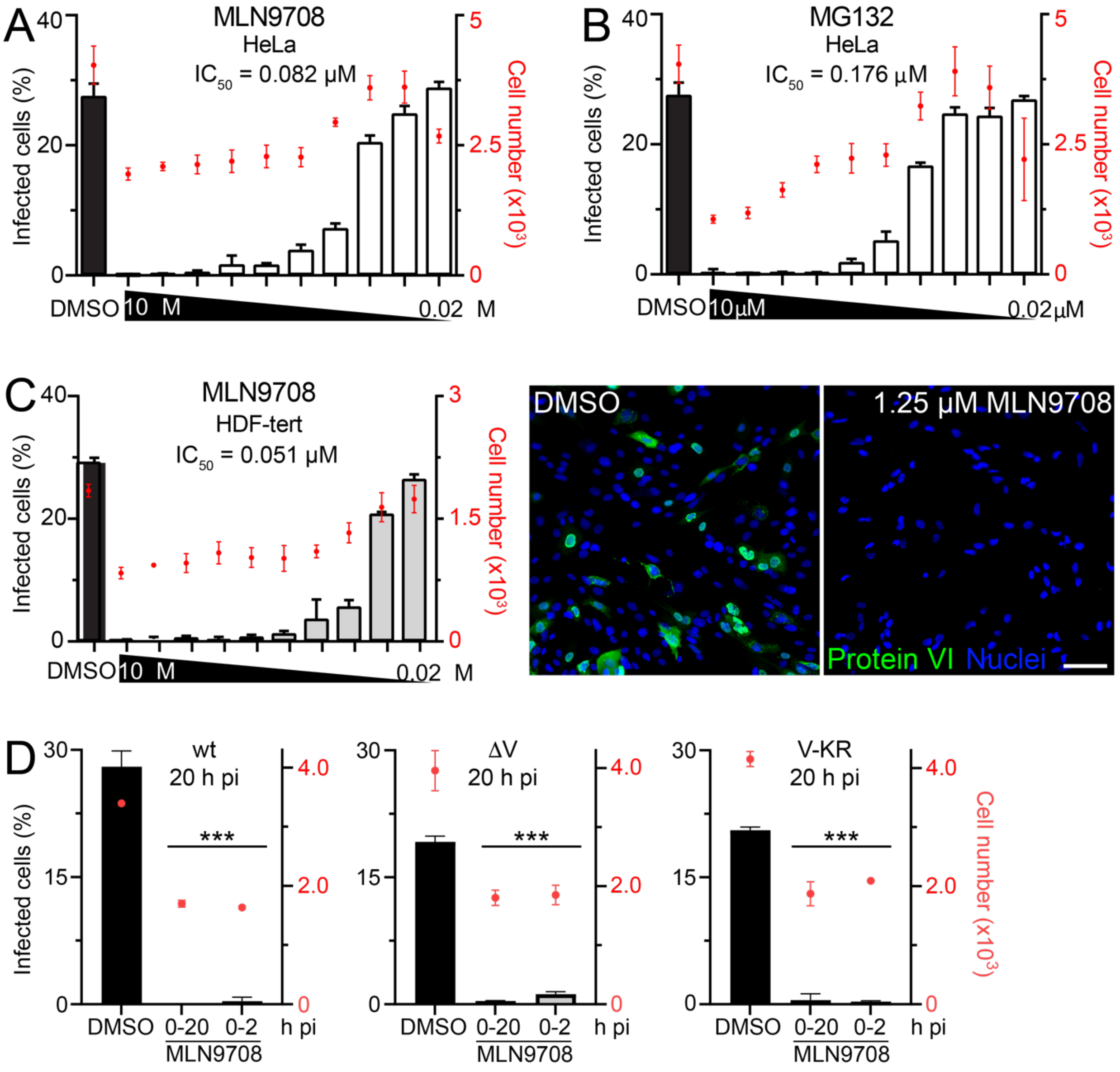

Fig. S5: Proteasome inhibition reduces AdV infection (refers to Fig. 7). (**A, B**) HeLa cells were infected with AdV-C5 at a MOI of 0.3 for 24h in the presence of DMSO or varying concentrations of proteasome inhibitors MLN9708 (A) or MG132 (B). After fixation, cells were stained with anti-pVI and DAPI. Infection was scored by percentage of pVI-positive nuclei. Data are shown as mean ± SD. (**C**) HDF-TERT cells were infected with AdV-C5 at a MOI of 0.3 for 24h in the presence of DMSO or varying concentrations of proteasome inhibitor MLN9708. Representative images are shown.

After fixation, cells were stained with anti-pVI antibody and DAPI. Infection was scored by percentage of pVI-positive nuclei. Scale bar, 100 μm. (**D**) Proteasome inhibition during the early phase of infection reduces AdV infection independent of protein V. HeLa cells were infected with AdV-C5, AdV-C5-ΔV or AdV-C5-V-KR at MOI 0.3 for 20h. MLN9708 (10 μM) was present for the entire infection (0-20h), only during entry (0-2h). Cells were fixed and processed as in (A). Data show the means ± SD. Statistical significance was assessed using a one-way ANOVA with Holm-Sidak correction for multiple comparisons. ***, $p<0.001$.

Supplementary Tables

Table S1: Number of peptides identified by mass sprectrometry and high confidence ubiquitinated peptides detected in viral proteins (refers to Fig. 5).

AdV protein	Identified peptides before enrichment	High-confidence GG-containing Lysine residues
Hexon	11	
Penton base	7	K155, K351
Protein IIIa	17	
Protein V	11	K141, K178, K188, K285
Protein VI	4	K52, K57, K70, K72, K79, K102, K106, K131
Protein VII	7	
Protein VIII	6	
Protein IX	6	

Table S2: DNA sequences of oligonucleotides and virus mutants (refers to Fig. 6).

**Oligo name**	**DNA sequence** (homology sequences in *italics*)
GalK_dV_f	*TCGTACTGTTGTATGTATCCAGCGGCGGCGGCGCGCAACGAAGCT*CCTGTTGACAATTAATCATCGGCA
GalK_dV_r	*GCGGCAGGTGAGGGCCATATCTGCAAGAACCACAAAGACCGGCTT*CTCAGCACTGTCCTGCTCCTT
dV_f	*TCGTACTGTTGTATGTATCCAGCGGCGGCGGCGCGCAACGAAGCTAAGCCGGTCTTTGTGGTTCTTGCAGATATGGCCCTCACCTGCCGC*
dV_r	*GCGGCAGGTGAGGGCCATATCTGCAAGAACCACAAAGACCGGCTTAGCTTCGTTGCGCGCCGCCGCCGCTGGATACATACAACAGTACGA*
GalK_Vmut_f	*CGCGAGTCTGGTGACTTGGCACCCACCGTGCAGCTGATGGTACCC*CCTGTTGACAATTAATCATCGGCA
GalK_Vmut_r	*TGGCCGCACGCGGACCTCGGGCTCCAGCCCAGGTTCCACGGTCAT*CTCAGCACTGTCCTGCTCCTT
V-KR-K178R_f	*CGCGAGTCTGGTGACTTGGCACCCACCGTGCAGCTGATGGTACCC*AGGCGCCAGCGACTGGAAGATGTCTTGGAAAAA*ATGACCGTGGAACCTGGGCTGGAGCCCGAGGTCCGCGTGCGGCCA*
V-KR-K178R_r	*TGGCCGCACGCGGACCTCGGGCTCCAGCCCAGGTTCCACGGTCAT*TTTTTCCAAGACATCTTCCAGTCGCTGGCGCCT*GGGTACCATCAGCTGCACGGTGGGTGCCAAGTCACCAGACTCGCG*
V-KR-K188R_f	*CGCGAGTCTGGTGACTTGGCACCCACCGTGCAGCTGATGGTACCC*AAGCGCCAGCGACTGGAAGATGTCTTGGAAAGA*ATGACCGTGGAACCTGGGCTGGAGCCCGAGGTCCGCGTGCGGCCA*
V-KR-K188R_r	*TGGCCGCACGCGGACCTCGGGCTCCAGCCCAGGTTCCACGGTCAT*TCTTTCCAAGACATCTTCCAGTCGCTGGCGCTT*GGGTACCATCAGCTGCACGGTGGGTGCCAAGTCACCAGACTCGCG*
V-KR-K178/188R_f	*CGCGAGTCTGGTGACTTGGCACCCACCGTGCAGCTGATGGTACCC*AGGCGCCAGCGACTGGAAGATGTCTTGGAAAGA*ATGACCGTGGAACCTGGGCTGGAGCCCGAGGTCCGCGTGCGGCCA*
V-KR-K178/188R_r	*TGGCCGCACGCGGACCTCGGGCTCCAGCCCAGGTTCCACGGTCAT*TCTTTCCAAGACATCTTCCAGTCGCTGGCGCCT*GGGTACCATCAGCTGCACGGTGGGTGCCAAGTCACCAGACTCGCG*
Mouse_CCL2_f	AAGAAGCTGTAGTTTTTGTCACCA
Mouse_CCL2_r	TTCCTTCTTGGGGTCAGCAC
Mouse_CXCL2_f	CATAGCCACTCTCAAGGGCG
Mouse_CXCL2_r	TTGGTTCTTCCGTTGAGGGAC
Mouse_CCL5_f	ATATGGCTCGGACACCACTC
Mouse_CCL5_r	GTGACAAACACGACTGCAAGA
Mouse_Il-1α_f	CCATGATCTGGAAGAGACCATCC
Mouse_Il-1α_r	GACGAGCTTCATCAGTTTGTATCTC
Mouse_cGAS_f	CAGCTACCAAGATGCTGTCAAAG
Mouse_cGAS_r	GAAGTGTTACAGCAGGGCTTC
Mouse_HPRT_f	CTGGTGAAAAGGACCTCTCG
Mouse_HPRT_r	TGAAGTACTCATTATAGTCAAGGGCA

V-KR	*TCGTACTGTTGTATGTATCCAGCGGCGGCGGCGCGCAACGAAGCT*ATGTC<br>CAGACGCCGTATCCGCGAAGAGATGCTCCAGGTCATCGCGCCGGAGATCT<br>ATGGCCCCCCGCGGAGAGAAGAGCAGGATTACCGACCCCGAAGGCTACGT<br>CGGGTCCGTCGCCGACGGAGAGATGATGATGATGAACTTGACGACGAGGT<br>GGAACTGCTGCACGCTACCGCGCCCAGGCGACGGGTACAGTGGAGGGGT<br>CGACGCGTACGTCGTGTTTTGCGACCCGGCACCACCGTAGTCTTTACGCC<br>CGGTGAGCGCTCCACCCGCACCTACCGCCGCGTGTATGATGAGGTGTACG<br>GCGACGAGGACCTGCTTGAGCAGGCCAACGAGCGCCTCGGGGAGTTTGC<br>CTACGGACGACGGCATCGGGACATGCTGGCGTTGCCGCTGGACGAGGGC<br>AACCCAACACCTAGCCTAAGACCCGTAACACTGCAGCAGGTGCTGCCCGC<br>GCTTGCACCGTCCGAAGAAAGGCGCGGCCTACGTCGCGAGTCTGGTGACT<br>TGGCACCCACCGTGCAGCTGATGGTACCCCGCCGCCAGCGACTGGAAGAT<br>GTCTTGGAACGAATGACCGTGGAACCTGGGCTGGAGCCCGAGGTCCGCGT<br>GCGGCCAATCCGGCAGGTGGCGCCGGGACTGGGCGTGCAGACCGTGGAC<br>GTTCAGATACCCACTACCAGTAGCACCAGTATTGCCACCGCCACAGAGGG<br>CATGGAGACACAAACGTCCCCGGTTGCCTCAGCGGTGGCGGATGCCGCG<br>GTGCAGGCGGTCGCTGCGGCCGCGTCCAGAACCTCTACGGAGGTGCAAA<br>CGGACCCGTGGATGTTTCGCGTTTCAGCCCCCCGGCGCCCGCGCGGTTC<br>GAGGAGGTACGGCGCCGCCAGCGCGCTACTGCCCGAATATGCCCTACATC<br>CTTCCATTGCGCCTACCCCCGGCTATCGTGGCTACACCTACCGCCCCAGAA<br>GACGAGCAACTACCCGACGCCGAACCACCACTGGAACCCGCCGCCGCCG<br>TCGCCGTCGCCAGCCCGTGCTGGCCCCGATTTCCGTGCGCAGGGTGGCT<br>CGCGAAGGAGGCAGGACCCTGGTGCTGCCAACAGCGCGCTACCACCCCA<br>GCATCGTTTAA*AAGCCGGTCTTTGTGGTTCTTGCAGATATGGCCCTCACCT*<br>*GCCGC*
V-KRrev*	*TCGTACTGTTGTATGTATCCAGCGGCGGCGGCGCGCAACGAAGCT*ATGT<br>CCAGACGCCGTATCCGCGAAGAGATGCTCCAGGTCATCGCGCCGGAG<br>ATCTATGGCCCCCCGCGGAGAGAAGAGCAGGATTACCGACCCCGAA<br>GGCTACGTCGGGTCCGTCGCCGACGGAGAGATGATGATGATGAACTT<br>GACGACGAGGTGGAACTGCTGCACGCTACCGCGCCCAGGCGACGGG<br>TACAGTGGAGGGGTCGACGCGTACGTCGTGTTTTGCGACCCGGCACC<br>ACCGTAGTCTTTACGCCCGGTGAGCGCTCCACCCGCACCTACCGCCG<br>CGTGTATGATGAGGTGTACGGCGACGAGGACCTGCTTGAGCAGGCCA<br>ACGAGCGCCTCGGGGAGTTTGCCTACGGACGACGGCATCGGGACATG<br>CTGGCGTTGCCGCTGGACGAGGGCAACCCAACACCTAGCCTAAGACC<br>CGTAACACTGCAGCAGGTGCTGCCCGCGCTTGCACCGTCCGAAGAAA<br>GGCGCGGCCTACGTCGCGAGTCTGGTGACTTGGCACCCACCGTGCAG<br>CTGATGGTACCCAAGCGCCAGCGACTGGAAGATGTCTTGGAAAAAAT<br>GACCGTGGAACCTGGGCTGGAGCCCGAGGTCCGCGTGCGGCCAATCC<br>GGCAGGTGGCGCCGGGACTGGGCGTGCAGACCGTGGACGTTCAGAT<br>ACCCACTACCAGTAGCACCAGTATTGCCACCGCCACAGAGGGCATGG<br>AGACACAAACGTCCCCGGTTGCCTCAGCGGTGGCGGATGCCGCGGTG<br>CAGGCGGTCGCTGCGGCCGCGTCCAGAACCTCTACGGAGGTGCAAAC<br>GGACCCGTGGATGTTTCGCGTTTCAGCCCCCCGGCGCCCGCGCGGTTC<br>GAGGAGGTACGGCGCCGCCAGCGCGCTACTGCCCGAATATGCCCTAC<br>ATCCTTCCATTGCGCCTACCCCCGGCTATCGTGGCTACACCTACCGCC<br>CCAGAAGACGAGCAACTACCCGACGCCGAACCACCACTGGAACCCG<br>CCGCCGCCGTCGCCGTCGCCAGCCCGTGCTGGCCCCGATTTCCGTGCG<br>CAGGGTGGCTCGCGAAGGAGGCAGGACCCTGGTGCTGCCAACAGCG<br>CGCTACCACCCCAGCATCGTTTAA*AAGCCGGTCTTTGTGGTTCTTGCAG*<br>*ATATGGCCCTCACCTGCCGC*

Supplementary Movie

Movie S1: Discharge of GFP-V from AdV-C2 at the nuclear membrane (refers to Fig. 5).

HeLa-sgMib1 cells expressing mScarlet-Mib1 were incubated with AdV-C2-GFP-V-atto647 at 37°C for 30 min. The virus inoculum was removed, and cells were placed in a confocal spinning-disk microscope. Timestamps are in min:sec. Arrows indicate GFP-V dissociation events. Three capsids containing GFP-V are visible at the nucleus, of which the two particles on the left discharge their GFP-V. Scale bar, 10 μm.

Supplementary Data

Data S1: NGS DNA sequence data of AdV-C5 and AdV-C5-ΔV (refers to Fig. 1).

Purified genomic DNA from AdV-C5 and AdV-C5-ΔV particles were sequenced through non-paired, short read Illumina NovaSeq 6000. Reads were mapped to AC_000008.1 reference using a Bowtie2 algorithm. Variant calling of the mapped reads was obtained with a mpileup algorithm using AC_000008.1 as reference.

Data S2: Mass spectrometry raw data (refers to Fig. 5).

Mass spectrometry data containing all identified AdV proteins following di-glycine enrichment. Data were generated from three independent biological replicates. Fold changes were calculated in $\log_2$.
